# Co-Targeting FASN and mTOR Suppresses Uveal Melanoma Growth

**DOI:** 10.3390/cancers15133451

**Published:** 2023-06-30

**Authors:** Anna Han, Dzmitry Mukha, Vivian Chua, Timothy J. Purwin, Manoela Tiago, Bhavik Modasia, Usman Baqai, Jenna L. Aumiller, Nelisa Bechtel, Emily Hunter, Meggie Danielson, Mizue Terai, Philip B. Wedegaertner, Takami Sato, Solange Landreville, Michael A. Davies, Stefan Kurtenbach, J. William Harbour, Zachary T. Schug, Andrew E. Aplin

**Affiliations:** 1 Department of Pharmacology, Physiology, and Cancer Biology, Thomas Jefferson University, Philadelphia, PA 19107, USA; annahan8659@jbnu.ac.kr (A.H.); vivian.chua@jefferson.edu (V.C.); timothy.purwin@jefferson.edu (T.J.P.); manoela.tiagodossantos@jefferson.edu (M.T.); usman.baqai@students.jefferson.edu (U.B.); emily.hunter@students.jefferson.edu (E.H.); 2 Department of Food Science and Human Nutrition, Jeonbuk National University, Jeonju 54896, Jeollabuk-do, Republic of Korea; 3 Molecular and Cellular Oncogenesis Program, The Wistar Institute, Philadelphia, PA 19104, USA; dmukha@wistar.org (D.M.); zschug@wistar.org (Z.T.S.); 4 Department of Biochemistry and Molecular Biology, Thomas Jefferson University, Philadelphia, PA 19107, USA; jenna.aumiller@students.jefferson.edu (J.L.A.); philip.wedegaertner@jefferson.edu (P.B.W.); 5 Department of Medical Oncology, Thomas Jefferson University, Philadelphia, PA 19107, USA; meggie.danielson@jefferson.edu (M.D.); mizue.terai@jefferson.edu (M.T.); takami.sato@jefferson.edu (T.S.); 6 Department of Ophthalmology and Otorhinolaryngology-Cervical-Facial Surgery, Faculty of Medicine, Université Laval, Québec, QC G1V 0A6, Canada; solange.landreville@fmed.ulaval.ca; 7 Department of Melanoma Medical Oncology, The University of Texas MD Anderson Cancer Center, Houston, TX 77030, USA; mdavies@mdanderson.org; 8 Bascom Palmer Eye Institute, University of Miami Miller School of Medicine, Miami, FL 33101, USA; stefan.kurtenbach@med.miami.edu (S.K.); william.harbour@utsouthwestern.edu (J.W.H.); 9 Sylvester Comprehensive Cancer Center, University of Miami Miller School of Medicine, Miami, FL 33101, USA; 10 Interdisciplinary Stem Cell Institute, University of Miami Miller School of Medicine, Miami, FL 33101, USA; 11 Department of Ophthalmology, Harold C. Simmons Comprehensive Cancer Center, University of Texas Southwestern Medical Center, Dallas, TX 75390, USA; 12 Sidney Kimmel Cancer Center, Thomas Jefferson University, Philadelphia, PA 19107, USA

**Keywords:** fatty acid synthase, mTOR pathway, metabolic inhibition, uveal melanoma, GNAQ

## Abstract

**Simple Summary:**

Metastatic uveal melanoma is often difficult to treat due to the lack of effective treatment options. Cancer cells rewire their metabolic features to support their energy needs for tumor growth and progression, and therefore targeting metabolic pathways may be a potential therapeutic approach in uveal melanoma. We aimed to identify unique metabolic features between uveal melanoma and normal uveal melanocytes and found that uveal melanoma cells expressed elevated levels of enzymes involved in lipid/fat metabolism such as fatty acid synthase (FASN). This was also associated with activation of the mTOR pathway. We then determined that inhibitors of FASN and mTOR led to the suppression of uveal melanoma cell growth. Our findings identified metabolic features that are unique in uveal melanoma compared to normal uveal melanocytes. Targeting of these features can lead to inhibition of cell growth and hence may be considered as a novel approach for the treatment of uveal melanoma.

**Abstract:**

Uveal melanoma (UM) displays a high frequency of metastasis; however, effective therapies for metastatic UM are limited. Identifying unique metabolic features of UM may provide a potential targeting strategy. A lipid metabolism protein expression signature was induced in a normal choroidal melanocyte (NCM) line transduced with *GNAQ* (Q209L), a driver in UM growth and development. Consistently, UM cells expressed elevated levels of fatty acid synthase (FASN) compared to NCMs. FASN upregulation was associated with increased mammalian target of rapamycin (mTOR) activation and sterol regulatory element-binding protein 1 (SREBP1) levels. FASN and mTOR inhibitors alone significantly reduced UM cell growth. Concurrent inhibition of FASN and mTOR further reduced UM cell growth by promoting cell cycle arrest and inhibiting glucose utilization, TCA cycle metabolism, and de novo fatty acid biosynthesis. Our findings indicate that FASN is important for UM cell growth and co-inhibition of FASN and mTOR signaling may be considered for treatment of UM.

## 1. Introduction

Cancer cells are highly plastic and may acquire malignant properties by rewiring metabolic pathways [1]. Targeting unique metabolic features of cancer cells provides a promising therapeutic strategy, especially for cancer types without effective treatments. Cancer cells display upregulated de novo lipogenesis to support energy storage, membrane generation, and cellular signaling [2,3]. While most normal human cells take up exogenous fatty acids (FAs), cancer cells frequently utilize de novo FA biosynthesis [4,5]. This pathway is controlled by lipogenic metabolic enzymes, including ATP-citrate lyase (ACLY), acetyl-CoA carboxylase (ACC) and fatty acid synthase (FASN), which are upregulated in some cancer cells [6,7]. Additional studies indicate that lipogenic metabolism of cancer cells can be a targetable vulnerability [2,8]. 

FASN is a critical enzyme in de novo FA biosynthesis and its overexpression is associated with poor prognosis and chemotherapy resistance in cancer [2,9,10]. The transcription factor sterol regulatory element-binding protein 1 (SREBP1) regulates the expression of lipogenic enzymes such as FASN [11]. In turn, SREBP1 is controlled by activation of mammalian target of rapamycin (mTOR), one of the major signaling nodes in cancer [12,13]. The mTOR–SREBP1 axis provides crosstalk between oncogenic signaling and cancer metabolism, and co-inhibition of metabolism and oncogenic signaling pathways is a strategy to improve treatment efficacy [1,6,10]. Such co-targeting regimens are likely to be particularly important in cancer types that have poor responses to current targeted therapies and immune checkpoint inhibitors. 

Uveal melanoma (UM) is the most common ocular melanoma in adults [14,15]. Although primary UM tumors are seemingly successfully treated with radiation therapy and surgery, about 50% of patients develop metastasis [14,16]. Mutations in G-protein coupled receptor pathway genes (*GNAQ*/*GNA11*) initiate UM development, whereas BRCA1-associated protein 1 (*BAP1*) mutations and monosomy 3 are associated with aggressiveness and metastasis of UM [17,18,19]. Despite these advances, therapeutic options for advanced-stage UM are limited [20]. Furthermore, targeted therapies and immune checkpoint inhibitors, which have markedly improved outcomes in cutaneous melanoma (CM) patients, have shown little activity in UM [14,17,21,22,23]. 

Gene expression profiling of metastatic UM tumors displays upregulated lipid metabolism, including glycerolipid and FA metabolism, compared to non-metastatic UM tumors [24]. Moreover, anchorage-independent growth of UM cells alters its metabolism toward lipogenesis [25]. However, the unique metabolic features of UM compared to normal choroidal melanocytes (NCMs) and its targetable vulnerabilities have not been fully investigated [26]. While monosomy 3/*BAP1* mutant-associated metabolic alterations have been described [27,28,29,30,31], the contribution of initiating *GNAQ/11* driver mutations is not known. Defining the metabolic features of UM will be critical to providing new therapeutic options for UM patients.

Here, we show that expression of lipid metabolic enzymes are elevated in an NCM cell line transduced with *GNAQ* (Q209L); these enzymes were also validated to be elevated in *GNAQ*/*11* mutant UM cells compared to NCMs. Upregulation of the key lipogenic enzyme, FASN, is associated with activation of the mTOR–SREBP1 axis. Inhibition of FASN alone significantly reduces UM cell growth; co-targeting FASN and mTOR further inhibits cell growth by increasing cell cycle arrest. Metabolic analysis showed that glucose utilization, the TCA cycle and de novo FA biosynthesis of UM cells are suppressed by combined targeting of FASN and mTOR. These findings highlight the importance of FASN for UM cell survival and the potential of co-targeting metabolic and oncogenic signaling pathways as a therapeutic strategy in UM.

## 2. Materials and Methods

### 2.1. Cell Culture and Generation of Cell Lines

Cell culture and generation of cell lines: UM001, MM66 and OMM1.3 (derived from a liver metastasis), UM004 (derived from orbital metastasis), 92.1 and MP46 (derived from primary tumor) cells have been previously described [28,32]. UMp006 and UMp007 were established from a PDX mouse model at Thomas Jefferson University. Sanger sequencing confirmed all cells to harbor *GNAQ* Q209 mutations. UM001 cells were cultured in RPMI-1640 media containing 10% heat-inactivated FBS (HI-FBS), 1% non-essential amino acids (NEAA), 1% L-glutamine and 1% HEPES buffer. UM004 cells were cultured in MEM supplemented with 10% HI-FBS and 1% L-glutamine. Cells of 92.1 were maintained in RPMI-1640 media supplemented with 10% HI-FBS and 1% L-glutamine, OMM1.3 cells were cultured in RPMI-1640 media supplemented with 10% FBS, and MP46 and MM66 cells were cultured in RPMI-1640 media containing 20% FBS. The A375 cutaneous melanoma cell line and HEK293 cells were grown in DMEM containing 10% FBS. The HEK293 GNAQ/11 knockout cells were generously provided by Dr. Asuka Inoue and Tohoku University (Sendai, Japan). GNAQ/11 knockout cells were generated using the CRISPR/Cas9 system [33]. All cell growth media contained 50 IU penicillin and 50 μg/mL streptomycin. UMp006 and UMp007 cells were cultured with Eagle’s Minimum Essential Medium supplemented with 20% FBS, 100 IU penicillin, and 100 µg/mL streptomycin. Cell lines were routinely tested for mycoplasma and authenticated by STR analysis. The most recent STR analysis was conducted in February 2021. 

### 2.2. Single-Cell RNA Analysis

UM scRNA Seq data from 11 patient tumor samples were obtained from the GEO database (accession GSE139829). The Seurat software package (v3.1.4) was used to process the data [34]. Cell types were identified using methods previously described [35]. Data were normalized using the SCTransform method [36] with regression based on the percentage of mitochondrial content. The ggplot2 software package (v3.3.2 https://ggplot2.tidyverse.org, accessed on 31 July 2020) was used to generate plots. Data analyses were performed in R (v4.0.2 http://www.R-project.org/, accessed on 31 July 2020).

### 2.3. Isolation of Human Choroidal Melanocytes

The cell lysates of normal human choroidal melanocytes (MCN#1459 and MCN#1462) were provided by Dr. Solange Landreville (Université Laval, Quebec City, Canada). Melanocytes were isolated from the choroid of two donor eyeballs (59 and 62 years old; Centre Universitaire d’ophtalmologie’s (CUO) Eye Bank, Quebec City, Canada) using an established protocol [37,38]. Isolated choroidal melanocytes (early passages, P2–P6) were cultured in mixed media of Ham’s F12 (45%) and DMEM medium (45%) supplemented with 10% FBS, 10 ng/mL cholera toxin (Sigma-Aldrich, St. Louis, MO, USA), 100 nM phorbol 12-myristate 13-acetate (Sigma-Aldrich), 100 µg/mL geneticin (Wisent, Quebec City, QC, Canada) and 50 µg/mL gentamicin (Gibco, Waltham, MA, USA). These NCMs are not immortalized but lysates were generated at the earliest passages before they became senescent. 

### 2.4. Analysis of Mass Spectrum Data

Lysates of UMC026 NCMs treated with doxycycline to induce GNAQ (Q209L) or GFP and mass spectrometry protein expression data from these cells were provided by Dr. Stefan Kurtenbach (University of Miami Miller School of Medicine, Miami, FL, USA). The UMC026 line (immortalized) was isolated from unaffected normal intraocular uveal tissue of a patient undergoing enucleation; the isolation process and culture conditions have been previously described [39,40]. Human-specific metabolism pathway entries from the KEGG pathway database [41] were collapsed into gene sets based on their metabolic subtype class. The total number of unique genes for the 10 collapsed sets ranged from 24 to 322. Protein expression levels for GNAQ (pQ209L) and GFP samples were scaled to total protein and log2-transformed with a pseudo-count of one. Proteins without expression in a sample were removed. The differences between GNAQ (pQ209L) and GFP log2 values were used for performing the GSEA pre-ranked method [42,43]. GSEA was performed on the collapsed KEGG metabolic pathway gene set collection using the weighted enrichment statistic parameter. Due to extreme peaks in the ranked protein list, we also used the classical method and subsequently omitted further investigation into sets matching known false-positive profiles [43].

### 2.5. Tissue Specimens

Formalin-fixed paraffin-embedded sections were collected from stage IV UM patients. This study was reviewed and approved by the Institutional Review Board at Thomas Jefferson University. 

### 2.6. Inhibitors

Fasnall (Sigma-Aldrich), GSK2194069, Fatostatin (Tocris, Minneapolis, MN, USA) and AZD2014 (Selleck Chemicals, Houston, TX, USA) were purchased, and the inhibitors were dissolved in DMSO. 

### 2.7. Short-Interfering RNA (siRNA) Transfection

Cells were seeded in 6-well plates overnight before transfection with chemically synthesized siRNAs at a final concentration of 25 nM using Lipofectamine™ RNAiMAX (Invitrogen, Carlsbad, CA, USA) as previously described [29]. FASN-specific siRNAs #1, UGACAUCGUCCAUUCGUUU, and #2, GAAGCACAUUGGCAAAGUC, were purchased from Dharmacon (Lafayette, CO, USA). GNAQ-specific siGENOME siRNAs #2, GCAACAAGAUGUGCUUAGA, and #3, GCAAGGCUCUCUUUAGAAC, were used. A non-targeting siRNA (UAGCGACUAAACACAUCAAUU) was used as a control.

### 2.8. Cell Growth Assay

Cells were seeded in 6-well plates for treatments. Growth was measured through crystal violet staining assay or IncuCyte imaging analysis (Essen Bioscience, Ann Arbor, MI, USA). For crystal violet staining, the cells were washed with PBS and stained with 0.2% crystal violet in 10% buffered formalin for 2–3 h. Plates were scanned and 100× magnification pictures were taken by the Nikon Eclipse Ti-e microscope and NIS-Elements AR 3.00 software (Nikon, Tokyo, Japan). These high-magnification images were analyzed using Image J to quantitate crystal violet staining [44]. For IncuCyte imaging analysis, images of cells were taken every 2 h and the occupied area (% confluency) was calculated using IncuCyte 2016B zoom software. 

### 2.9. Reverse Phase Protein Array (RPPA)

Cells were treated as outlined in figure legends, lysed [45] and analyzed at the MD Anderson Functional Proteomic core facility (Houston, TX, USA). Data analysis was performed as described [32]. Comparisons between conditioned samples were performed using the two-sample *t*-test method with 1000 permutations. Multiple hypothesis test corrections were calculated, and antibodies with a Storey q-value < 0.05 and a fold ratio > 25% were considered significant. Calculations were conducted in MATLAB (v2017b) using the mattest and mafdr functions. 

### 2.10. Annexin V/Propidium Iodide (PI) Apoptosis Assay

Cells were trypsinized, and cell pellets were washed twice with PBS and then re-suspended in binding buffer containing 1:20 annexin V-APC (BD Pharmingen, San Jose, CA, USA) and 0.2 mg/mL PI (Life Technologies; Carlsbad, CA, USA). Samples were incubated at room temperature for 15 min, protected from light. Annexin V-APC and PI fluorescence was analyzed using flow cytometry. 

### 2.11. EdU Cell S Phase Entry Assay

Cells cultured in 6-well plates were treated as indicated. Sixteen hours prior to the end of treatment, 10 µM EdU was added to cultures and cells then processed using the Click-iT^TM^ Plus EdU Alexa Fluor^TM^ 647 flow cytometry assay kit (Thermo Fisher Scientific, Waltham, MA, USA), following the company’s protocol.

### 2.12. Tumor 3D Spheroid Formation

The detailed protocol is described in [46]. In brief, 92.1 cells (2.5 × 104) were plated on a 1.5% agar bed (*w*/*v*) for 5 days. Three-dimensional spheroids were transferred and embedded into collagen I solution. The next day, collagen-embedded 3D tumor spheroids were treated as described in figure legends. For cell viability, cells were incubated with calcein-AM (7 µM) and propidium iodide (PI, 10 μg/mL) for 30 min at the end of the experiment. Pictures were obtained using a Nikon A1R confocal microscope. For EdU incorporation, cells were incubated with analogue EdU (10 µM) for 16 h. Tumor spheroids were extracted from collagen solution using collagenase type 1 (Sigma-Aldrich) and EdU incorporation was measured using the Click-iT^TM^ Plus EdU Alexa Fluor^TM^ 647 flow cytometry assay kit (Thermo Fisher Scientific), following company instructions. 

### 2.13. Immunohistochemistry (IHC) and Scoring

Formalin-fixed paraffin-embedded tissue sections of the liver from metastatic UM patients were de-paraffinized in a series of xylenes and graded ethanol. Antigen retrieval was performed by a heat-mediated method for 20 min in a vegetable steamer in 10 mM sodium citrate buffer (pH 6.0) for FASN and S100. Slides were washed in a tris-buffered saline (TBS) with 0.025% Tween 20. Tissue sections were blocked with the Bioxall endogenous peroxidase and alkaline phosphatase solution (Vector Laboratories, Burlingame, CA, USA) for 20 min followed by 10% normal horse serum for 2 h before primary antibody incubation (overnight at 4 °C). Then, slides were washed using TBS with 0.1% Tween 20 and blocked using anti-rabbit IgG ImmPRESS UNIVERSAL (Vector Laboratories) for 1 h. The color was developed using ImmPACT VECTOR RED AP (Vector Laboratories). Tissue sections were counterstained using Hematoxylin (Vector Laboratories). The following primary antibodies were used: FASN (#3180) from Cell Signaling Technology (Danvers, MA, USA) and S100 (#Z0311) and Rabbit IgG isotope control from DAKO/Currently Aligent (Santa Clara, CA, USA). 

The intensity of positively expressed area was measured using a Nikon Eclipse 50i microscope with NIS-Elements D3.1 software (Nikon). Staining was evaluated by a pathologist and total intensity was scored by adding coverage score and the cumulative intensity score [47]. Briefly, coverage score was measured as 0, none; 1,1/100–1/10; 2, 1/10–1/3; 3, 1/3–2/3 and 4, 2/3 to 100%. The intensity score was measured (0, 1, 2 or 3) and calculated by creating a range based on the cumulative intensity score across all positively expressed tumor areas. 

### 2.14. Western Blotting

Cell lysates were prepared in Laemmli sample buffer containing β-mercaptoethanol. Proteins were resolved by SDS-PAGE and transferred to PVDF. Membranes were blocked in 1% BSA and incubated in primary antibodies overnight at 4 °C. Proteins were detected using HRP secondary antibodies and chemiluminescence substrate (Pierce, Rockford, IL, USA) on a ChemiDoc Imaging System (Bio-Rad, Hercules, CA, USA). The following primary antibodies were used: BAP1 (#13271), Aurora-A (#3092), PLK1 (#4513), cyclin B1 (#4135), Wee1 (#4936), phospho-RB (#9308), S6 (#2217), PKM2 (#3198), GLUT1 (#12939), HK1 (#2024), HK2 (#2867), PKM2 (#3198), FASN (#3180), ACC (#3662), ACSL1 (#9189), ACLY (#4332), CPT1A (#12252), phospho-ACC (S79, #3661), phospho-mTOR (S2448, #2971), mTOR (#2983), phospho-ERK1/2 (#9101), ERK1/2 (#9102), phospho-S6 (S235/236, #4857), phospho-S6 (S240/244, #2215), and HSP90 (#4877) from Cell Signaling Technology; CPT1C (ab123794) from Abcam (Cambridge, UK); GLUT3 (sc-30107) and GNAQ (sc-393) from Santa Cruz Biotechnology, Inc. (Dallas, TX, USA); SREBP1 (#557036) from BD Pharmingen and β-actin (#A2066) from Sigma-Aldrich. 

### 2.15. Metabolomics and Lipidomics

Chemicals and reagents: Cells plated in separate Petri dishes were counted prior to extraction for the calculation of consumption and secretion rates. Cells were washed once with PBS pH 7.4. Metabolism was quenched by addition of 1.5 mL of MeOH. Cells were lifted with a cell scraper. Both methanol extract and cell pellet were transferred to 2 mL centrifuge tubes. To each tube, 375 µL of water was added. The tubes were vortexed and then centrifuged at 17,000× *g* for 30 min at 4 °C; 375 µL of the supernatant was transferred to new tubes and the centrifugation was repeated, while the rest was used for lipid extraction. After the second centrifugation, 40 µL of metabolite extract was transferred to the LCMS vials. 

Hydrophilic metabolite analysis: Chromatographic separation was achieved on a SeQuant ZIC-pHILIC column (2.1 × 150 mm, 5 μm, EMD Millipore, Burlington, MA, USA). Flow rate was set to 0.2 mL∙min^−1^, column compartment was set to 30 °C, and autosampler tray maintained at 4 °C. Mobile phase A consisted of 20 mM ammonium carbonate and 0.01% (*v*/*v*) ammonium hydroxide. Mobile Phase B was 100% acetonitrile. The mobile phase linear gradient (%B) was as follows: 0 min 80%, 15 min 20%, 15.1 min 80%, 23 min 80%. A mobile phase was introduced to a Thermo Q Exactive HF-X mass spectrometer (Thermo Fisher Scientific) with an electrospray ionization source working in polarity switching mode. Ionization source parameters were the following: sheath gas 40, auxiliary gas 10, spray voltage −3.25 kV or +4.25 kV, capillary temperature 325 °C, S-lens RF level 50 and auxiliary gas temperature 50 °C. Metabolites were analyzed in the range of 70–1000 m/z. Positions of metabolites in the chromatogram were identified by corresponding pure chemical standards. Data were analyzed in MAVEN [48]. 

Free fatty acid extraction: From the remaining cell extract, 1 mL was discarded without perturbing cell pellet. Then, cell extract and cell pellet were transferred to glass tubes, and 4 mL of chloroform:methanol 3:1 containing 20 µL of 5% Avanti EquiSplash per sample were added (final 2:1 chloroform:methanol). Tubes were vortexed ~10 s each, and then incubated at 37 °C for 30 min. An amount of 800 µL PBS was added to induce phase separation. Samples were vortexed and centrifuged at 3000× *g* 1 min at room temperature. The lower phase was aspirated with Pasteur glass pipettes (using a pipette filler) and transferred to new glass tubes. The chloroform phase was evaporated under nitrogen flow. The organic solvent phase was evaporated in the nitrogen flow. Lipid extract was re-dissolved in 1 mL of 0.3 M KOH in 90% methanol and incubated in a water bath at 80 °C for 1 h. After cooling the tubes, saponification was quenched with 100 mL of formic acid. Fatty acids were extracted with 800 µL of hexane. The organic phase was evaporated in the nitrogen flow. The pellet was re-dissolved in 200 µL of 50:50 isopropanol:methanol; 10 µL were used per injection.

LC-MS analysis of free fatty acids: Analyte separation was achieved with a Kinetex XB-C18 column (2.6 µm beads, 150 × 3 mm). The column compartment of HPLC was maintained at 65 °C and sample vials at 30 °C. The mobile phase was composed of the two solvent mixtures: A—60:40 acetonitrile:water with 10 mM ammonium formate, B—90:8:2 isopropanol:acetonitrile:water with 10 mM ammonium formate. The buffer loop in the autosampler was filled with a mix of 50:50 isopropanol:methanol (95% of the final volume) and 5% water supplemented with formic acid (0.02% final concentration). Solvent flow was maintained at 0.5 mL/min during the whole run. Mobile phase composition was changing according to the following program (in respect to solvent B): 0 min—15%; 3 min—60%; 8 min—82%; 8.5 min—95%; 11 min—100%, 15 min—100%; 15.1 min—15%, maintained until 17.5 min. Chromatographic eluent was ionized in electrospray ion source HESI-II and analyzed by Thermo Orbitrap Q Exactive HF-X mass spectrometer. The ion source was supplied with 50 arbitrary units (AU) of sheath gas, 20 AU auxiliary gas, and no spare gas flow. Auxiliary gas and the ion transfer capillary were heated to 350 °C. S-Lens RF level was 80 AU. The analysis was performed in polarity switching mode with the negative mode voltage at −3.5 kV and the positive mode voltage at +3.3 kV. The automatic gain control (AGC) target was set to 5∙106, the scanning range was from 120 to 1800 *m*/*z* and the mass resolution was 60,000 in both polarity modes. Data were analyzed in MAVEN.

### 2.16. Statistical Analysis

The data from the cell viability experiments was presented as mean and standard error of the mean of at least three biological replicates. For RPPA analysis, four biological replicates of each cell line were utilized. The analyses of 13C-glucose trace experiments were conducted using six biological replicates. Statistical significance was calculated using the unpaired *t*-test and/or two-way ANOVA with Dunnett’s multiple comparison test.

## 3. Results

### 3.1. UM Cells Have Elevated Lipogenic Enzyme Expression

**Figure 1:**
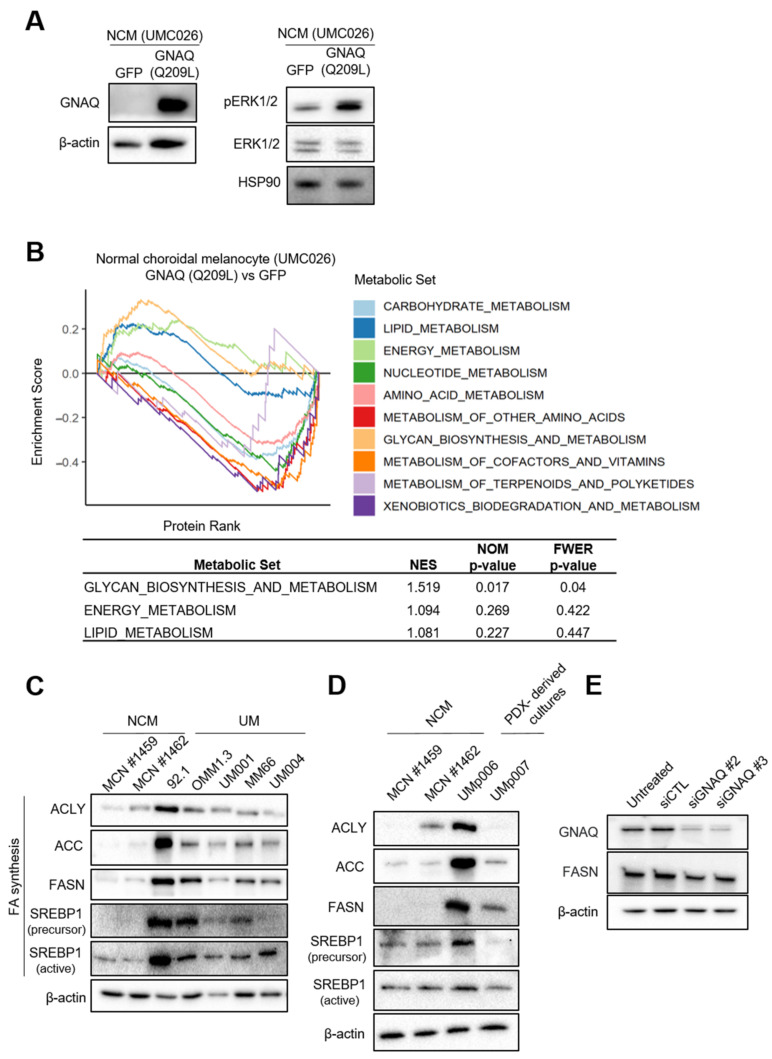
Increased FASN expression in UM cells. (**A**) GNAQ and pERK1/2 levels in UMC026 cells transduced with exogenous *GNAQ* (Q209L) shown by Western blot. GNAQ antibody detects all forms of GNAQ. (**B**) An enrichment plot of GSEA results using the weighted enrichment statistic parameters. (**C**) Expression of major lipogenic enzymes in NCMs (MCN#1459 and MCN#1462) and UM cell lines were evaluated by Western blot. (**D**) The levels of ALCY, ACC, FASN and SREBP1 in PDX-derived UM cell lines compared to NCMs. (**E**) FASN levels following *GNAQ* knockdown by siRNA transfection for 72 h in MP46 cells. β-actin or HSP90 served as loading controls. NCMs; normal choroidal melanocytes, ACLY; ATP-citrate lyase, ACC; acetyl-CoA carboxylase, FASN; fatty acid synthase, SREBP1; sterol regulatory element-binding protein and PDX; patient derived xenografts.

To identify how a *GNAQ* mutation affects metabolism, we performed pathway enrichment analysis on mass spectrometry protein expression data from an NCM cell line, UMC026, following doxycycline-inducible expression of exogenous *GNAQ* (Q209L) or GFP (control). We performed a Western blot to show elevated levels of GNAQ and phosphorylated ERK1/2 (pERK1/2), which indicates an increase in MAPK signaling activity following *GNAQ* (Q209L) transduction (Figure 1A and Figure S8). Others have also shown that the MAPK signaling pathway becomes activated following *GNAQ* (Q209L) expression [49,50,51]. We analyzed the human-specific metabolism pathway entries from the Kyoto Encyclopedia of Genes and Genomes (KEGG) database [41] using the gene set enrichment analysis (GSEA) method [42]. We observed that transduction of mutant *GNAQ* led to positive enrichment of lipid metabolism as well as energy metabolism and glycan biosynthesis and metabolism pathways, while carbohydrate metabolism was negatively enriched (Figure 1B and Figure S1A,B). We next compared the expression of major enzymes involved in FA and glucose metabolism between two NCMs (MCN#1459 and MCN#1462) and UM cells. While UM cells did not show dramatic differences in metabolic enzymes related to glucose uptake, glycolysis, or FA oxidation (Figures S2A and S8), UM cells exhibited increased levels of several enzymes involved in the lipogenic pathway, including ACLY, ACC and FASN, compared to NCMs (Figure 1C and Figures S2B and S8). UM cells also displayed upregulated SREBP1 expression, a key lipogenic transcription factor compared to NCMs (Figure 1C and Figures S2B and S8). FASN levels were also higher in patient-derived xenograft (PDX) cell cultures (Figure 1D and Figures S2C and S8). Consistently, knockdown of *GNAQ* by siRNA transfection decreased FASN levels in MP46, a UM cell line harboring *GNAQ* Q209L (Figure 1E and Figures S2D and S8). ACLY, ACC and FASN expression were not affected by BAP1 status (Figures S2E and S8). We compared the effects of transducing WT *GNAQ* to mutant *GNAQ* in HEK293 *GNAQ*/*11* knockout cells. *GNAQ* WT moderately increased levels of FASN but transduction with mutant *GNAQ* (Q209L and Q209P) markedly increased FASN expression levels (Figures S2F and S8). Notably, this is consistent with an increase in phosphorylation of ERK1/2 in cells expressing mutant *GNAQ* but not in cells expressing *GNAQ* WT. Overall, our findings show that lipid metabolism and enzymes associated with the lipogenic pathway such as FASN are induced in UM and by mutant *GNAQ*.

**Figure 2:**
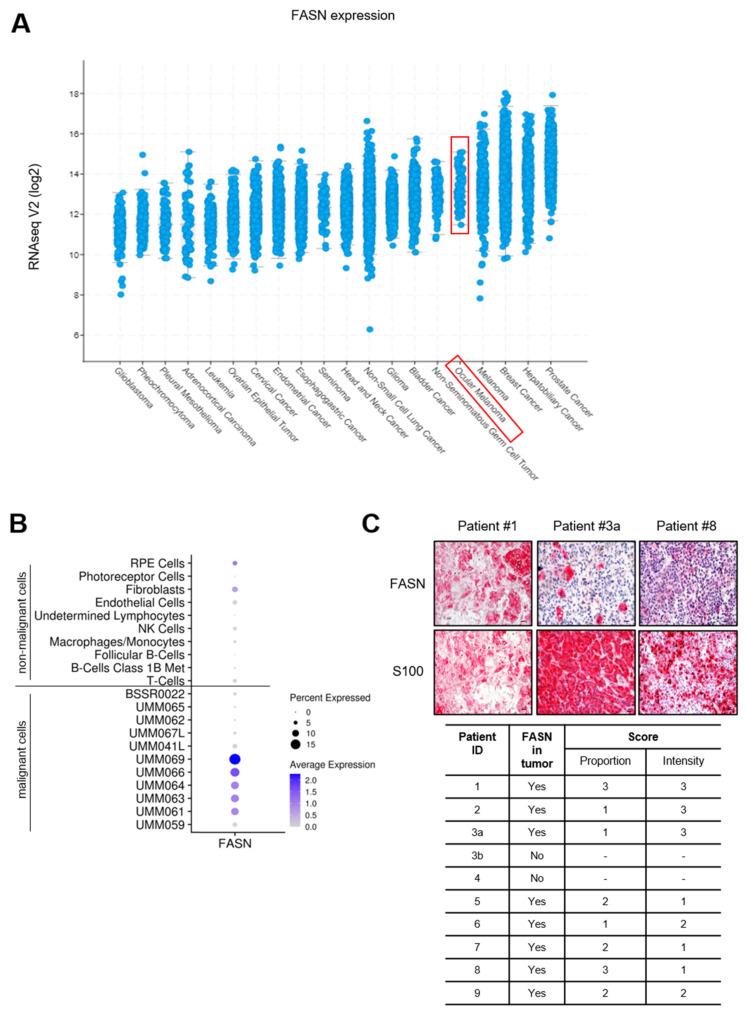
Elevated FASN expression in UM patient sample. (**A**) Gene expression of FASN in various human cancer types. Data were derived from a TCGA dataset and analyzed through the Firebrowse web resource (http://firebrowse.org, accessed on 24 February 2021). Red box indicates uveal melanoma samples (UM). (**B**) Dot plot showing the average expression and percent of cells expressing FASN from patient tumor scRNA-seq data. Cells were separated into non-malignant and tumor-specific malignant cell groups. BSSR0022, UMM041L and UMM067L were isolated from metastases whereas the other malignant cases were from primary tumors. (**C**) FASN expression in liver metastasized UM tumors. S100 was detected as marker for melanoma cells. Scale bar: 10 µm.

We analyzed FASN and SREBP1 gene expression across several cancer types by analyzing the Cancer Genome Atlas (TCGA) dataset. This analysis showed that UM is within the top five of cancers expressing high levels of FASN and SREBP1 (Figure 2A and Figure S3A). The other top five cancers include breast cancer, melanoma and hepatobiliary cancer, which harbor few to no mutations in *GNAQ* [52]. Hence, while we show that mutant *GNAQ* regulates FASN expression in UM, there are other factors that can also regulate FASN in UM and other cancer types. Since the TCGA dataset lacks a comparison with normal tissue, we analyzed single-cell RNA sequencing from the GEO database (accession GSE139829). We observed that a subset (five of eleven cases) of malignant cells have higher expression of FASN and SREBP1 compared to non-malignant cells (Figure 2B and Figure S3B). In addition, we analyzed UM liver metastases from patients and observed marked FASN expression in the tumor tissue area (Figure 2C).

### 3.2. Co-Targeting FASN and mTOR Represses the Growth of UM Cells

**Figure 3:**
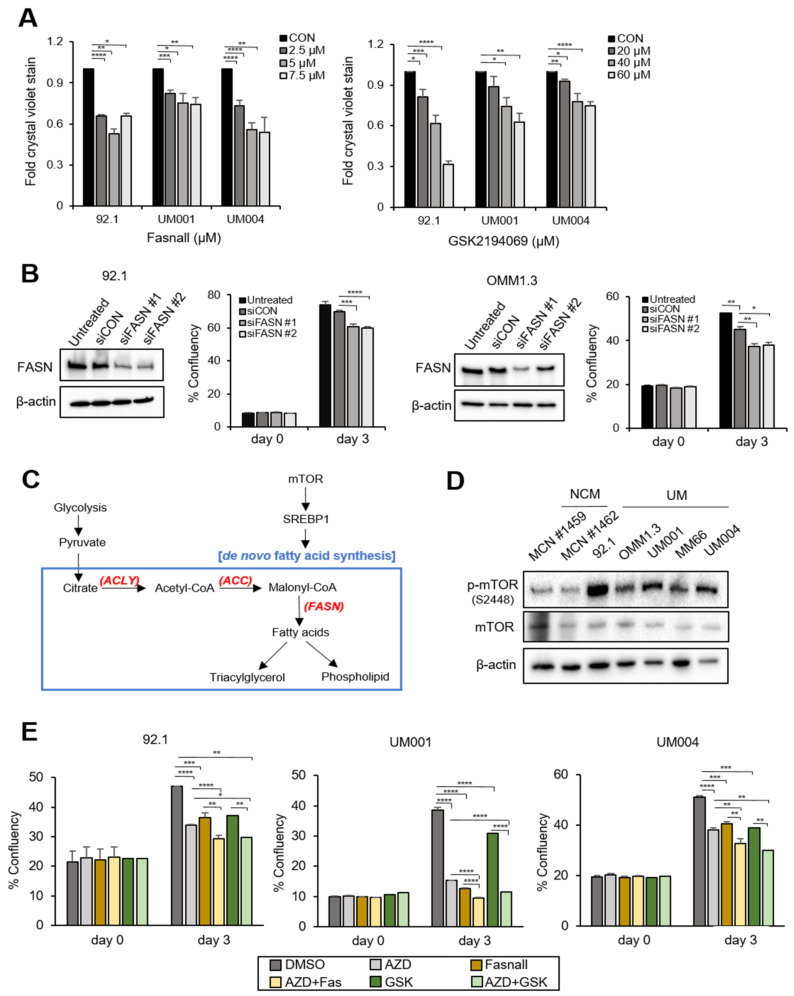
Co-targeting FASN and mTOR reduces UM cell growth. (**A**) 92.1, UM001 and UM004 cells were treated with Fasnall (0, 2.5, 5 and 7.5 μM) for 4 days or GSK 2194069 (0, 20, 40 and 60 μM) for 3 days. Cell viability was measured by crystal violet staining. Quantification of cell growth following treatment with Fasnall and GSK2194069 is shown as fold changes in crystal violet stain compared to controls. (**B**) FASN knockdown was performed by siRNA transfection. Silencing of *FASN* was confirmed through Western blot. (**C**) Activation of mTOR modulates de novo lipogenesis in cancer cells by controlling the expression of transcription factor (SREBP1) and key lipogenic enzymes (e.g., ACLY, ACC and FASN; colored red). (**D**) Phosphorylated and total mTOR levels in NCMs and UM cell lines were probed by Western blot. β-actin served as a loading control. (**E**) The effects of co-inhibition of FASN and mTOR in 92.1, UM001 and UM004 cell growth were measured by IncuCyte. The cells were treated with Fasnall (5 μM) or GSK2194069 (40 μM), with or without AZD2014 (200 nM), for 72 h. Percent confluency of the cells was measured on day 0 and day 3. Data are shown as mean ± SEM (n = 4) * *p* < 0.05, ** *p* < 0.01, *** *p* < 0.001 and **** *p* < 0.0001 unpaired *t*-test. NCMs; normal choroidal melanocytes, mTOR; mammalian target of rapamycin, ACLY; ATP-citrate lyase, ACC; acetyl-CoA carboxylase, FASN; fatty acid synthase and SREBP1; sterol regulatory element-binding protein.

Targeting elevated FASN inhibits cancer cell growth in HER2-positive breast cancer and glioma stem cells [53,54]. To determine the requirement of FASN in UM cell growth, we treated *GNAQ* mutant UM cells (Figure S4A) with the FASN inhibitors Fasnall and GSK2194069 [55]. We observed that both FASN inhibitors significantly suppressed UM cell growth (Figure 3A and Figure S4B). Moreover, knockdown of FASN also significantly reduced UM cell viability (Figure 3B and Figure S8). 

Expression of key lipogenic enzymes, including FASN, is often regulated by the mTOR-SREBP1 axis in cancer cells (Figure 3C) [56,57]. We compared mTOR activity between NCMs and UM cells and observed that UM cells have markedly elevated levels of phosphorylated mTOR (Figure 3D and Figure S8). This finding suggests that the mTOR pathway is activated in UM cells. Next, we tested whether FASN expression is controlled by the mTOR-SREBP1 axis in UM cells. UM cells were treated with an mTOR inhibitor (AZD2014) or SREBP1 inhibitor (Fatostatin). Treatment with AZD2014 reduced the levels of SREBP1 precursor and lipogenesis-associated proteins, including FASN and ACC, in UM001 and OMM1.3 cells (Figures S4C and S8). In addition, Fatostatin decreased expression of precursors SREBP1 and FASN in UM001 cells (Figures S4D and S8). These observations show that FASN expression in UM cells is regulated by the mTOR–SREBP1 axis. To test the effects of co-inhibition of FASN and mTOR, we treated UM cells with Fasnall or GSK2194069, with or without the mTOR inhibitor AZD2014. We also tested an mTORC1 inhibitor, rapamycin, as an alternate mTOR inhibitor in UM004 cells. As expected, mTOR inhibition alone significantly diminished cell growth (Figure 3E and Figure S5A). Additionally, co-inhibition of FASN and mTOR elicited modest but statistically enhanced growth inhibitory effects compared to either treatment alone (Figure 3E and Figure S5A). These results suggest that co-targeting FASN and mTOR holds therapeutic potential in UM. We also tested the effects of the FASN and mTOR inhibitors on a non-GNAQ mutant cutaneous melanoma cell line, A375. The mTOR inhibitors AZD2014 and rapamycin alone elicited modest reduction in A375 cell viability (Figure S5B). The FASN inhibitor Fasnall induced a modest decrease in cell viability as well, but GSK2194069 significantly inhibited A375 cell growth. The combinations of FASN and mTOR inhibitors did not further decrease cell viability of A375 cells compared to single-agent treatment (Figure S5B). 

### 3.3. Inhibition of FASN and mTOR Alters Cell Cycle Regulator Expression in UM Cells

**Figure 4:**
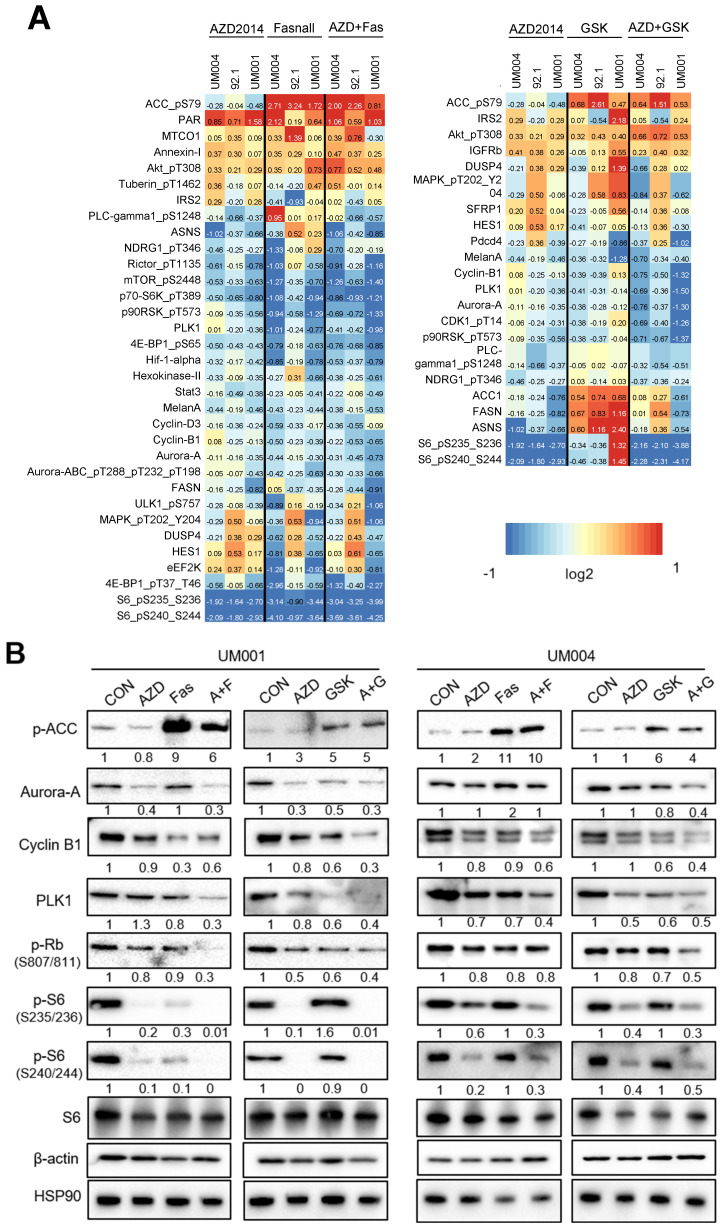
Effects of FASN and mTOR inhibitors on levels of cell cycle modulators in UM. UM004, 92.1 and UM001 cells were treated with Fasnall (5 μM) or GSK2194069 (40 μM), with or without AZD2014 (200 nM), for 48 h. (**A**) RPPA data were used to determine proteins/phospho-proteins that were significantly different between control, single treatments of each inhibitor and combo treatments of different cell lines (*p*-value < 0.05 and a 25% log2 fold change). Comparisons were performed between each group using the two-sample *t*-test method with 1000 permutations and assumed unequal variance. Hierarchical clustering was performed based on median-centered log2-transformed expression values. Statistical calculations were performed in Matlab^®^ (v2015b) using the mattest function. (**B**) Results of RPPA analysis were validated by Western blot in UM001 and UM004 cells. β-actin, HSP90 and S6 served as loading controls. Protein expression was normalized to the average intensity of the loading controls.

To characterize the anti-growth effects of FASN and mTOR inhibitors in UM, we performed reverse phase protein array (RPPA) analysis. RPPA results were confirmed by Western blot. Fasnall and GSK2194069 (FASN inhibitors) and/or AZD2014 (mTOR inhibitor) decreased the expression of proteins that are associated with cell cycle progression, including aurora-A, polo-like kinase 1 (PLK1), cyclin B1 and phospho-RB1 (S807/811), and cell growth, including phospho-S6 (S235/236 and S240/244) (Figure 4A,B and Figure S8). Both FASN inhibitors suppress the β-ketoacyl reductase activity of FASN, and Fasnall does not directly target the acetyl-CoA carboxylase (ACC) protein [10]. We observed that both FASN inhibitors increased phosphorylation/inactivation of ACC in UM cells (Figure 4A,B and Figure S8). Overall, these data suggest that FASN and mTOR inhibitors’ effects on UM cell growth are associated with cell cycle suppression.

### 3.4. Inhibition of FASN and mTOR Reduce Cell Growth through Cell Cycle Arrest in UM Cells

**Figure 5:**
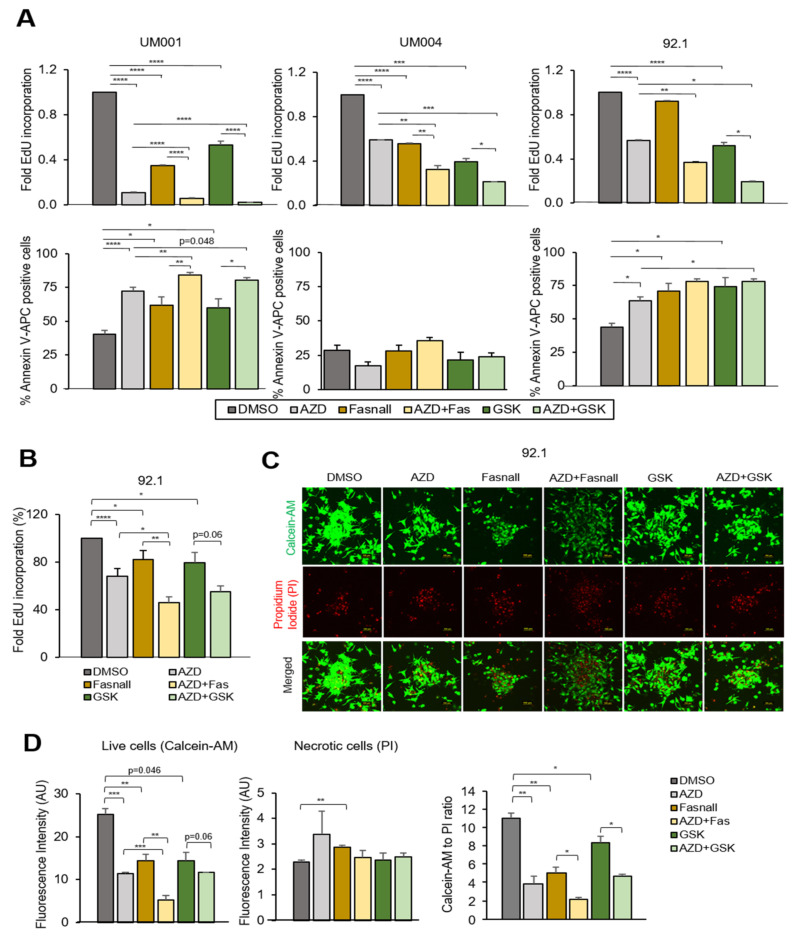
Inhibition of FASN and mTOR induce cell cycle arrest in 2D and 3D cell growth. UM004, 92.1 and UM001 cells were treated with Fasnall (5 μM) or GSK2194069 (40 μM), with or without AZD2014 (200 nM), for 48 h. (**A**) Cells were collected for EdU corporation (upper panel) and annexin/PI staining assays (bottom panel). (**B**) 3D spheroid cultures of 92.1 cells were treated with Fasnall (5 μM) or GSK2194069 (40 μM), with or without AZD2014 (200 nM), for 48 h. EdU incorporation of 92.1 grown as 3D spheroids is shown. (**C**) Representative figures of spheroids are shown. Tumor spheroids were treated with Fasnall (5 μM) or GSK2194069 (40 μM), with or without AZD2014 (200 nM), for 48 h. For cell viability, 3D spheroids were stained with Calcein-AM and PI for live cells and necrotic cells, respectively. (**D**) Quantitation of Calcein-AM and PI. Magnification: 150X, Scale bar: 100 µm. Quantification bar graph is shown. Data are shown as mean ± SEM (n = 4). * *p* < 0.05, ** *p* < 0.01, *** *p* < 0.001 and **** *p* < 0.0001 unpaired *t*-test.

Since targeting FASN and mTOR decreased expression of cell cycle regulators, we performed EdU incorporation assays. Both FASN inhibitors and the mTOR inhibitor significantly reduced the EdU incorporation percentage, indicative of S-phase entry inhibition (Figure 5A, upper panel). Co-treatment of FASN and mTOR inhibitors further decreased EdU incorporation. Additionally, we observed that both FASN inhibitors and the mTOR inhibitor induced apoptosis individually, and co-inhibition further increased apoptosis in 92.1 and UM001 cells (Figure 5A, bottom panel).

To mimic a more physiological setting, we cultured 92.1 cells as 3D spheroids and treated them with FASN and mTOR inhibitors. Consistent with the results of 2D cultures, individual treatment of FASN and mTOR inhibitors significantly decreased EdU incorporation into 92.1 cells as 3D spheroids, and co-inhibition further suppressed EdU incorporation (Figure 5B). We observed consistent results with Calcein-AM staining of 3D spheroid of 92.1 (Figure 5C,D). These observations indicate that a combination of FASN and mTOR inhibitors reduces UM cells growth by inducing cell cycle arrest and in some cell lines, increasing apoptosis as well.

### 3.5. FASN and mTOR Inhibitors Diminish Fatty Acid Biosynthesis in UM Cells

**Figure 6:**
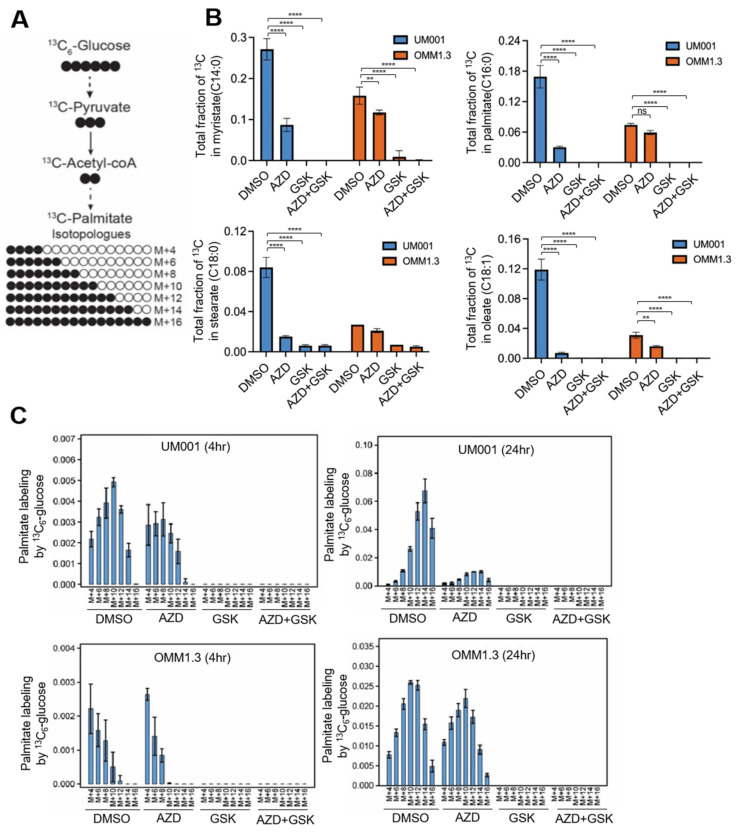
Suppression of FASN and mTOR decrease de novo FA biosynthesis in UM cells. UM001 and OMM1.3 cells were incubated in the presence of 13C-glucose for 4 and 24 h along with GSK2194069 (40 μM) and with or without AZD2014 (200 nM). (**A**) A flow of carbon atoms from the glucose to the fatty acid palmitate, including depictions of M + 4 through M + 16 isotopologues. Black circles = carbon-13; white circles = carbon-12. (**B**) Fractional labeling of myristate (14:0), palmitate (16:0), palmitoleate (16:1) and stearate (18:0) in UM cells at 24 h after the addition of 13C-glucose. Data are shown as mean ± SD (n = 3). ns, not significant, ** *p* < 0.01, and **** *p* < 0.0001 Two-way ANOVA with Dunnett’s multiple comparison testing. (**C**) Isotopologue distribution patterns of palmitate from 13C-glucose in UM001 and OMM1.3 cells at 4 h (left panel) and 24 h (right panel). Data are shown as mean ± SD (n = 3).

To further investigate the on-target effects of FASN inhibitors and the overall impact of FASN and mTOR inhibitors on UM metabolism, an LC-MS-based carbon-13 (13C) tracer approach was employed (Figure 6A). UM001 and OMM1.3 cells were incubated in the presence of uniformly labeled 13C-glucose. The effects of an FASN inhibitor (GSK2194069) and mTOR inhibitor (AZD2014) on de novo FA biosynthesis were analyzed. There were no differences detected in inhibitor uptake between the cells (Figure S6). As determined by the incorporation of a 13C-glucose label into myristate (C14:0), palmitate (C16:0), stearate (C18:0) and oleate (C18:1), UM001 cells exhibited an overall higher rate of FA biosynthesis compared to OMM1.3 cells (Figure 6B). In-depth analysis of palmitate isotopologue distribution patterns showed that GSK2194069 effectively eliminates FA biosynthesis in UM001 and OMM1.3 cells but that AZD2014 elicited partial effects (Figure 6C).

### 3.6. Suppression of FASN and mTOR Impairs Glucose Utilization and TCA Cycle Metabolism in UM Cells

**Figure 7:**
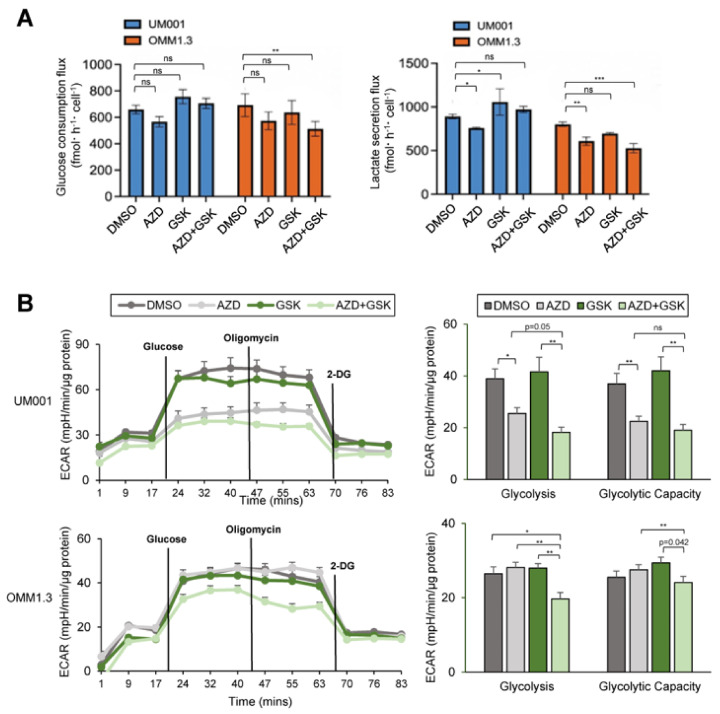
The effects of FASN and mTOR inhibitors on glucose utilization in UM cells. UM001 and OMM1.3 cells were incubated in the presence of 13C-glucose for 4 and 24 h along with GSK2194069 (40 μM) and with or without AZD2014 (200 nM). (**A**) Net flux of glucose consumption and lactate secretion from UM cells calculated at fmol/hour/cell. Data are shown as mean ± SD (n = 3). ns, not significant, * *p* < 0.05, ** *p* < 0.01, and *** *p* < 0.001. Two-way ANOVA with Dunnett’s multiple comparison testing. (**B**) Glycolytic capacity of UM001 and OMM1.3 cells after the treatment (24 h) was measured by ECAR using the Seahorse analyzer. Data were normalized to protein level and analyzed via Agilent Seahorse XF report generators. Data are shown as mean ± SEM (n = 12). ns, not significant, * *p* < 0.05, and ** *p* < 0.01.

FASN also plays a critical role in overall metabolic remodeling of cancer cells [58,59]. To elucidate whether inhibition of FASN and mTOR influences other metabolic pathways in UM cells, we calculated the net flux of glucose and lactate into the culture media of UM001 and OMM1.3 cells using 13C-glucose tracing. AZD2014 alone suppressed glucose uptake and lactate secretion in both UM001 and OMM1.3 cells (Figure 7A). The combination of GSK2194069 and AZD2014 further decreased glucose uptake and lactate secretion in OMM1.3 cells, but not in UM001 cells (Figure 7A). To confirm these changes, we analyzed the glycolytic capacity of UM001 and OMM1.3 cells after the inhibitor treatment using the Seahorse XF analyzer. AZD2014 markedly decreased glycolysis and glycolytic capacity in UM001 cells, but not in OMM1.3 cells (Figure 7B). However, the combination of GSK2194069 and AZD2014 significantly suppressed glycolysis and glycolytic capacities in both cell lines relative to single treatments (Figure 7B).

**Figure 8:**
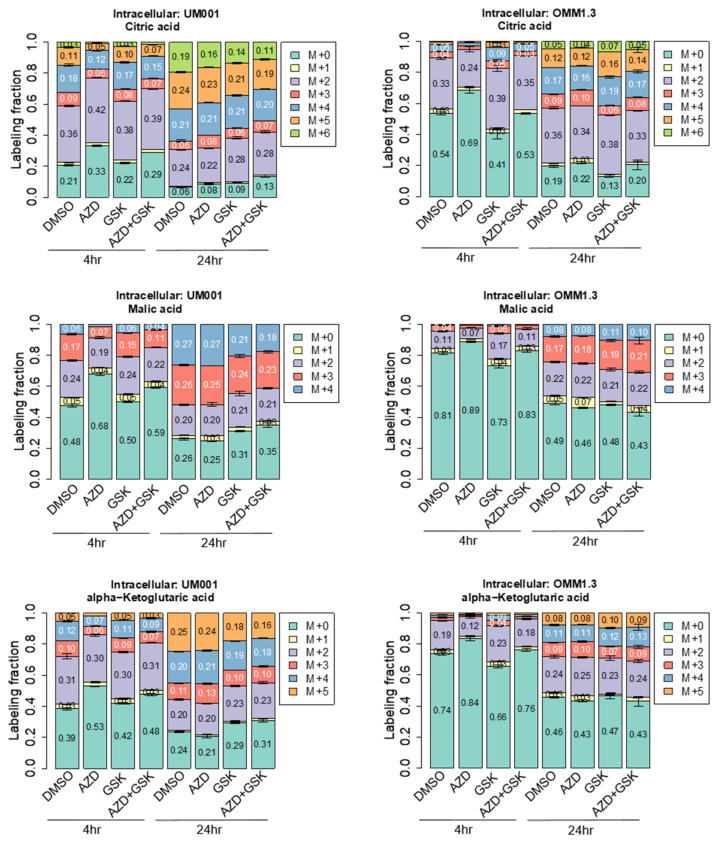
Inhibition of FASN and mTOR decrease the flux of glucose into the TCA cycle in UM cells. UM001 and OMM1.3 cells were incubated in the presence of 13C-glucose for 4 and 24 h along with GSK2194069 (40 μM) and with or without AZD2014 (200 nM). Fractional labeling of TCA cycle intermediates in UM001 and OMM1.3 cells (4 and 24 h) after the addition of 13C-glucose. Inset numbers indicate percent labeling for each isotopologue (n = 3).

We also measured 13C-glucose-dependent labeling of TCA cycle intermediates, including citrate, malate and α-ketoglutarate at 4 and 24 h to investigate the effect of FASN and mTOR inhibitors on the TCA cycle metabolism. AZD2014 inhibited 13C-labeling of TCA cycle intermediates in both cell lines, implying potent inhibition of mitochondrial metabolism. In contrast, FASN inhibition (GSK2194069) did not affect the flux of glucose carbon into the TCA cycle. Co-treatment with GSK2194069 and AZD2014 displayed an intermediate effect, but overall, the levels of 13C-glucose-labeled TCA cycle intermediates were decreased (Figure 8). These observations suggest that GSK2194069 might potentially impact TCA cycle metabolism due to the reduced demands for citrate and acetyl-CoA for FA biosynthesis. Moreover, when UM cells reached “steady-state” labeling from 13C-glucose at 24 h, there were no drastic differences in total levels of 13C-labeled TCA cycle intermediates (Figure 8), indicating that UM cells do not switch their nutrient source from glucose to another nutrient to support metabolism. Altogether, these observations indicate that co-targeting FASN and mTOR hinders glucose utilization, glucose flux into the TCA cycle and FA biosynthesis.

## 4. Discussion

In this study, we report that transduction of mutant *GNAQ*, which causes an initial event in UM and driver mutation in the development of UM, increased expression of enzymes involved in lipid metabolism in a NCM line. Consistent with this data, we also show that UM cells exhibit elevated FASN expression compared to NCMs and knockdown of *GNAQ* resulted in decreased FASN expression. Elevated FASN is regulated by the mTOR–SREBP1 axis. Inhibition of FASN and mTOR alone significantly suppressed UM cell growth. Co-targeting FASN and mTOR further inhibited cell growth by inducing cell cycle arrest and apoptosis, decreasing glucose utilization, TCA cycle metabolism and de novo FA biosynthesis of UM cells. 

Targeted inhibitor and immune checkpoint therapies have benefited a subset of CMs but elicit poor responses in UM [22,60], and therefore novel approaches are essential to broaden therapeutic options for advanced-stage UM. A recent study revealed that metastatic monosomy 3 UM has elevated mitochondrial functional capacity due to upregulated succinate dehydrogenase A levels compared to non-metastatic monosomy 3 [27]. These findings indicate that UM has reprogrammed cellular metabolism, which may present as a new therapeutic option for UM patients [26]. Relative to normal cells, cancer cells display altered metabolic characteristics, including increased aerobic glycolysis, nucleotide biosynthesis and de novo lipogenesis [1,5]. Thus, unique metabolic features of cancer cells that are not present in normal cells represent a targetable vulnerability. Furthermore, the effects of targeting unique cancer cell metabolism pathways have been investigated in other cancer types, including CM [61,62], but are poorly understood in UM. 

Activation of oncogenes and/or inactivation of tumor-suppressor genes leads to the metabolic alteration of cancer cells [63]. For instance, KIT mutations, one of the major mutations observed in mucosal melanoma (~25%), are related with the upregulation of glucose metabolism in mucosal melanoma cells [64,65]. *BAP1* mutations are strongly associated with metastasis of UM and regulate UM cellular metabolism, including energy regulatory signaling pathways [28], glycolysis [29], mitochondrial reserve activity [27] and metabolic heterogeneity [31]. While signaling pathways downstream of *GNAQ/GNA11* mutations are well studied, their effects on cellular metabolism are incompletely investigated [17]. We transduced an NCM line with mutant *GNAQ,* and due to our focus being on effects on metabolism-associated pathways, we do not report biological changes in the NCM line following transduction in this study. We show that the transduction of the UMC026 NCM cell line with mutant *GNAQ* altered several metabolic pathways, including upregulating expression of enzymes involved in lipid metabolism such as FASN and energy metabolism, as well as glycan biosynthesis and metabolism pathways. By contrast, *GNAQ* (Q209L) downregulates carbohydrate metabolism-related proteins in NCM. These findings were compared to cells transduced with a GFP control only and not to wild-type *GNAQ* as well. This is a limitation of our study. We were unable to generate UMC026 cells transduced with wild-type GNAQ in time for this study. Nevertheless, here we have determined whether levels of GNAQ were comparable between our melanocyte model and UM cell lines. GNAQ levels were higher in UMC026 transduced with *GNAQ* Q209L than in UM cell lines (Figure S7). Importantly, we have data for HEK293 cells showing that FASN levels were only moderately upregulated in HEK293 *GNAQ*/*11* knockout cells transduced with WT *GNAQ,* but mutant *GNAQ* markedly increased FASN levels. Additionally, mutant *GNAQ* increased pERK while WT *GNAQ* did not. This indicates that expression of mutant *GNAQ* and hyperactivation of downstream signaling such as the MAPK pathway induce FASN. Overall, these observations suggest that further understanding of the association between *GNAQ/GNA11* mutations and UM cellular metabolism may open new therapeutic avenues for UM patients. 

Using gene expression profiling and pathway analyses, several studies have reported that NCMs and UM might have different cellular metabolisms [26,66,67,68,69]. For example, NCMs showed elevated gene expression related to glycogen and amino acid metabolism, while UM cells displayed increased gene expression involved in glycolysis and mitochondrial respiration [66]. However, the potential of targeting UM-associated metabolic pathways has not been fully evaluated. Our findings showing that FASN inhibitors markedly reduce UM cell growth suggest that lipogenic metabolism is important to UM pathobiology and is a targetable vulnerability of UM. Further studies are necessary to assess the efficacy of FASN inhibition in an in vivo model. 

Metabolic enzyme inhibitors can also influence normal proliferative cells [70], and thus targeting metabolic enzymes involved in a deregulated oncogenic pathway may provide selectivity toward cancer cells [1]. Combination approaches that target cancer metabolism and oncogenic pathways may also prevent the likelihood of drug resistance. Targeting the mTOR pathway with targeted therapies (e.g., MEK inhibitors) has been studied in UM [71,72,73]; however, the roles of activated mTOR pathways in UM pathobiology and the effects of co-targeting mTOR and cellular metabolism have not been studied. We showed that activation of the mTOR–SREBP1 axis is responsible for increased lipogenic enzyme expression in UM. Furthermore, co-inhibition of mTOR and FASN further suppressed UM cell growth by inhibiting not only cell viability but also cellular metabolism compared to a single target approach, thereby suggesting new promising combinatorial options for UM patients. 

There are other limitations in this study. Lipogenesis is mainly regulated by SREBP1-mTOR complex 1 (mTORC1) axis; however, in this study, we used AZD2014, which inhibits both mTORC1 and mTORC2 [74,75]. We tested an mTORC1 inhibitor, rapamycin, in one cell line that elicited similar effects as AZD2014, and future studies may confirm this in multiple cell lines. Additionally, it will be desirable to perform a formal analysis of synergism or antagonism of the drug combinations in future studies. The concentrations of FASN inhibitors used in our in vitro experiments are in the µM range and may be a limitation by causing concerns for toxicity in in vivo or human settings. Metabolic inhibitors are frequently used at µM concentrations due to high cellular levels of target metabolites [76]. The high concentration used may cause pharmacokinetic issues, and it is necessary to consider this point when translating our findings to in vivo studies and/or clinical trials in the future. However, FASN inhibitors have been tested in vivo at concentrations that are well tolerated in mice and that also led to inhibition of tumor growth, findings which were consistent with results from in vitro experiments [53]. Additionally, our studies focused on expression or knockdown of *GNAQ*. Though we observed similar expression levels of lipogenic enzymes in *GNA11* mutant and *BAP1* mutant UM cells, it will be important in future studies to test the effects of targeting FASN and mTOR in these cells. 

## 5. Conclusions

In conclusion, this study offers evidence indicating that FASN plays an important role in UM cell growth. By incorporating bio-informatics, RPPA-based proteomics, metabolomics and molecular analyses, this study addresses whether co-targeting metabolic and oncogenic signaling pathways could be a new therapeutic strategy in UM. We demonstrate that transduction of mutant *GNAQ* is correlated with positive enrichment of protein expression involved in lipid metabolism in NCM. Compared to NCMs, UM cells displayed elevated lipogenic enzyme levels, including FASN, which can be regulated by an active mTOR–SREBP1 axis. Additionally, knockdown of *GNAQ* in UM reduced FASN expression levels. Although suppression of FASN alone strongly decreased the growth of UM cells, the co-suppression of FASN and mTOR further decreased UM cell growth. Co-inhibition of FASN and mTOR led to cell cycle arrest and/or apoptosis, and reduction of several metabolic pathways, including glucose metabolism, TCA metabolism and de novo FA biosynthesis in UM cells. Our findings suggest that co-targeting lipogenic phenotypes and activated oncogenic signaling pathways can be a promising and novel approach for UM. 

## Data Availability

Restrictions apply to the availability of these data. Data was obtained from co-authors and are available with the permission of our co-authors.

## Supplementary Materials

The following supporting information can be downloaded at: https://www.mdpi.com/article/10.3390/cancers15133451/s1, Figure S1: The effects of *GNAQ* mutation in the metabolism of NCM. (A) An enrichment plot of GSEA results using the classic enrichment statistic parameters is presented. (B) A list of downregulated GSEA results using the weighted and classic enrichment statistic parameters in presented; Figure S2: Major metabolic enzyme expression in NCMs and UM cells. (A) Expression of several key enzymes in glucose uptake, glycolysis, and fatty acid oxidation in NCMs and UM cells were probed by western blot. (B) Quantitation of expression of lipogenic enzymes in NCMs and UM cells shown in Figure 1C by densitometry. (C) Quantitation of expression of lipogenic enzymes in NCMs and PDXs shown in Figure 1D. (D) MP46 cells were treated with siRNA against *GNAQ* for 72 h. GNAQ and FASN expression were probed by western blot and quantitated by densitometry. β-actin serves as a loading control. Data are shown as mean ± SEM (n = 3). * *p* <0.05, ** *p* < 0.01 (E) The protein expression of major lipogenic enzymes in BAP1 wild-type and BAP1-deficient UM cell lines were evaluated by western blot. (F) Western blot of FASN in HEK293 *GNAQ*/11 knockout cells transduced with WT or mutant *GNAQ*. β-actin or HSP90 serve as loading controls. NCMs, normal choroidal melanocytes; GLUT1 and 3, glucose transporter; HK1 and 2, hexokinase; PKM2, pyruvate kinase-2; ACSL1, acyl CoA synthetase-1; and CPT1A and 1C, carnitine palmitoyltransferase1; Figure S3: Expression of SREBP1 in UM patient samples. (A) Gene expression of SREBP1 in various human cancer types. Data were derived from a TCGA dataset and analyzed through the Firebrowse web resource (http://firebrowse.org/, accessed on 24 February 2021). Box plots display FASN and SREBP1 levels measured as RSEM log2 across various cancers. Red box indicates uveal melanoma samples (UVM). (B) The average expression of SREBP1 in non-malignant and malignant cells from UM tumors; Figure S4: mTOR-SREBP1 axis regulates FASN expression in UM cells. (A) GNAQ mutational status of UM cells. (B) 92.1, UM001, and UM004 cells were treated with Fasnall (0, 2.5, 5 and 7.5 μM) for 4 days or GSK 2194069 (0, 20, 40 and 60 μM) for 3 days. Cell viability was measured by crystal violet staining. Representative images from four biological replicates are shown. (C) UM001 and OMM1.3 cells were treated with AZD2014 (0, 50, 100 and 200 nM) for 48 h. Activation of mTOR and expression of SREBP1, FASN, ACC and ACLY were detected by western blot. (D) UM001 cells were treated with Fatostatin (0, 5, 10 and 15 μM) for 24 h. Reduction of SREBP1 and FASN expression were probed by western blot. β-actin serves as a loading control. Data are shown as mean ± SEM (n = 3). ns, not significant, * *p* < 0.05, ** *p* < 0.01, *** *p* < 0.001 and **** *p* < 0.0001 unpaired *t*-test; Figure S5: FASN and mTOR inhibitor effects in non-GNAQ mutant and UM cell lines. (A) UM004 UM cells and (B) the A375 (non-GNAQ mutant) cutaneous melanoma cell line were treated with FASN inhibitors (5 µM Fasnall or 40 µM GSK2194069) and/or mTOR inhibitors (200 nM AZD2014 or 100 nM Rapamycin) for 72 h. Cell viability was determined by crystal violet staining. Data are shown as mean ± SEM (n = 3). * *p* < 0.05, ** *p* < 0.01 unpaired *t*-test. Representative crystal violet images are also shown; Figure S6: Uptake of FASN and mTOR inhibitors by UM cells. UM001 and OMM1.3 cells were incubated in the presence of ^13^C-glucose for 4 and 24 h along with GSK2194069 (40 μM) with or without AZD2014 (200 nM). Peak area intensity for AZD2014 and GSK2194069 in cells as determined by LC-MS/MS. Data are shown as mean ± SEM (n = 6). Student *t*-test; Figure S7: GNAQ levels in melanocyte model and UM cell lines. Western blot was performed to identify GNAQ levels between UMC026 cells transduced with *GNAQ* Q209L with UM cell lines, 92.1, OMM1.3, UM001, and UM004; Figure S8: Original western blots.
