# Supplementary material for: Co-Targeting FASN and mTOR Suppresses Uveal Melanoma Growth
Source: Cancers (Basel). 2023 Jun 30;15(13):3451. doi: 10.3390/cancers15133451 (PMC10341317; doi:10.3390/cancers15133451)
Supplement: Supplementary file 1 [file cancers-15-03451-s001.zip › cancers-2466949-supplementary.pdf]

A

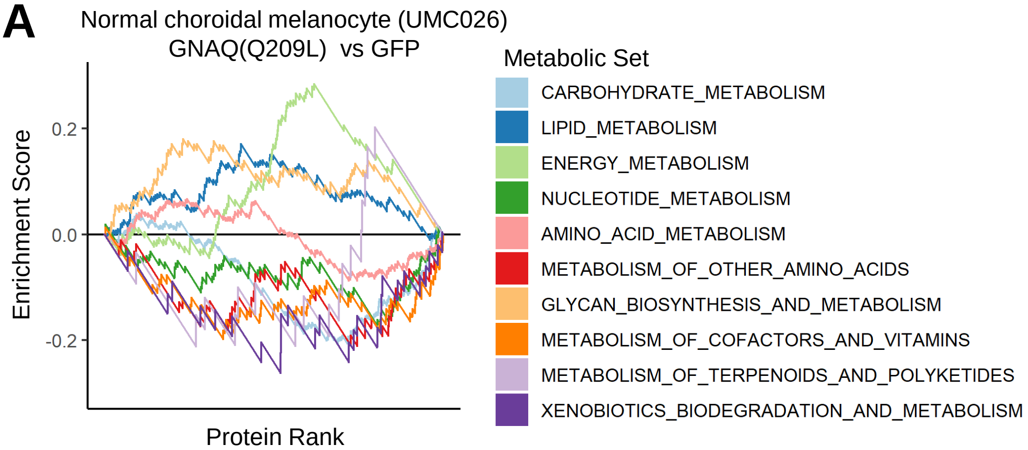

Metabolic Set	NES	NOM p-value	FWER p-value
ENERGY_METABOLISM	3.287	< 0.001	< 0.001
LIPID_METABOLISM	2.159	0.002	0.004
GLYCAN_BIOSYNTHESIS_AND_METABOLISM	2.015	0.004	0.021

B

Weighted results

Metabolic Set	NES	NOM p-value	FWER p-value
AMINO_ACID_METABOLISM	-1.317	0.042	0.083
CARBOHYDRATE_METABOLISM	-1.580	0.001	0.009
XENOBIOTICS_BIODEGRADATION_AND_METABOLISM	-1.603	0.017	0.009
NUCLEOTIDE_METABOLISM	-1.691	< 0.001	0.003
METABOLISM_OF_COFACTORS_AND_VITAMINS	-1.859	< 0.001	< 0.001
METABOLISM_OF_OTHER_AMINO_ACIDS	-1.871	< 0.001	< 0.001

Classic results

Metabolic Set	NES	NOM p-value	FWER p-value
XENOBIOTICS_BIODEGRADATION_AND_METABOLISM	-1.599	0.033	0.057
METABOLISM_OF_OTHER_AMINO_ACIDS	-1.784	0.023	0.030
METABOLISM_OF_COFACTORS_AND_VITAMINS	-1.853	0.014	0.025
NUCLEOTIDE_METABOLISM	-1.968	0.008	0.019
CARBOHYDRATE_METABOLISM	-3.088	<0.001	<0.001

Figure S1. The effects of *GNAQ* mutation in the metabolism of NCM.
(**A**) An enrichment plot of GSEA results using the classic enrichment statistic parameters is presented. (**B**) A list of downregulated GSEA results using the weighted and classic enrichment statistic parameters in presented.

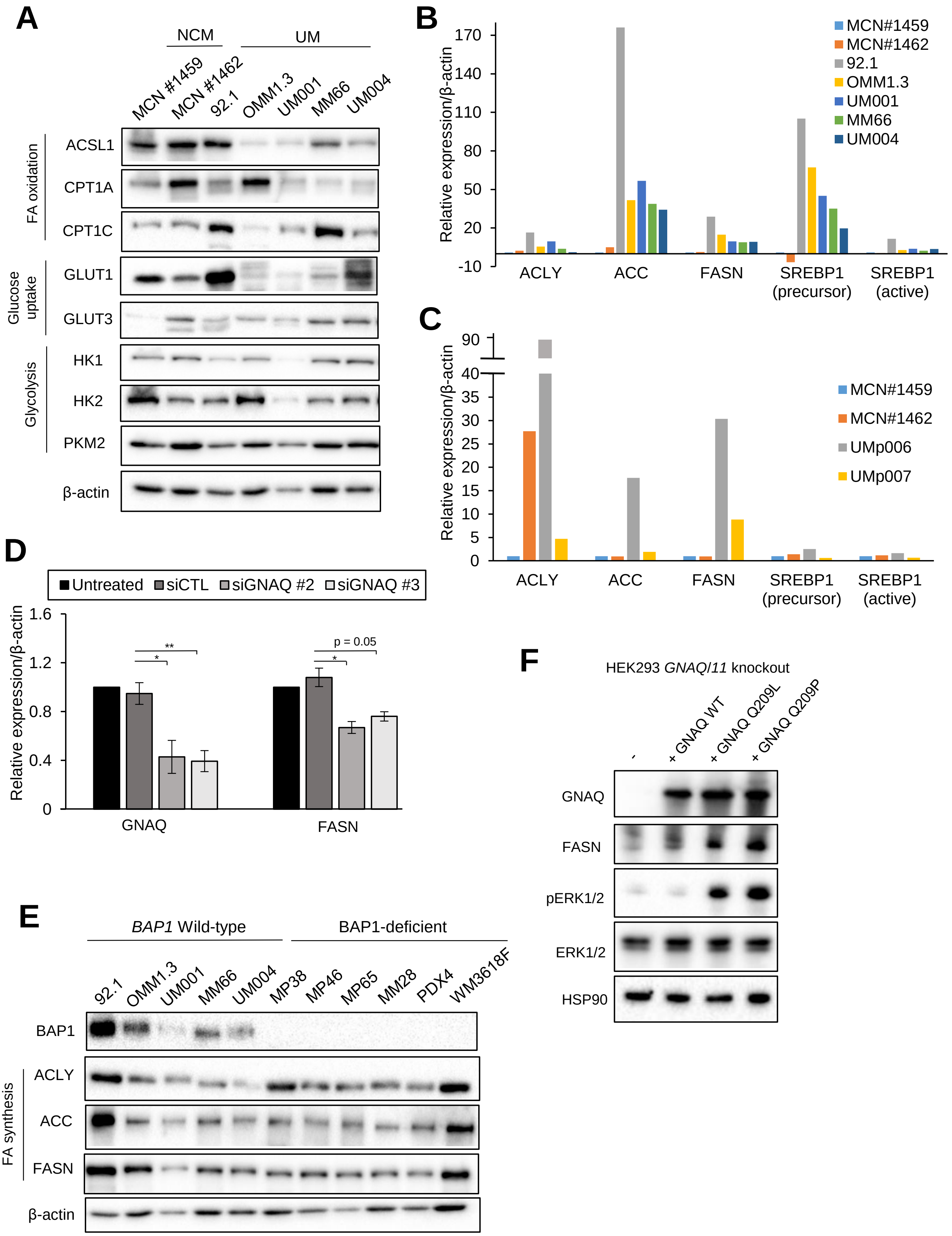
A
NCM
UM
MCN #1459
MCN #1462
92.1
OMM1.3
UM001
MM66
UM004
FA oxidation
ACSL1
CPT1A
CPT1C
Glucose uptake
GLUT1
GLUT3
Glycolysis
HK1
HK2
PKM2
β-actin
B
Relative expression/β-actin
170
140
110
80
50
20
-10
MCN#1459
MCN#1462
92.1
OMM1.3
UM001
MM66
UM004
ACLY
ACC
FASN
SREBP1 (precursor)
SREBP1 (active)
C
Relative expression/β-actin
90
40
35
30
25
20
15
10
5
0
MCN#1459
MCN#1462
UMp006
UMp007
ACLY
ACC
FASN
SREBP1 (precursor)
SREBP1 (active)
D
Untreated
siCTL
siGNAQ #2
siGNAQ #3
Relative expression/β-actin
1.6
1.2
0.8
0.4
0
**
*
p = 0.05
*
GNAQ
FASN
E
BAP1 Wild-type
BAP1-deficient
92.1
OMM1.3
UM001
MM66
UM004
MP38
MP46
MP65
MM28
PDX4
WM3618F
BAP1
FA synthesis
ACLY
ACC
FASN
β-actin
F
HEK293 GNAQ/11 knockout
-
+ GNAQ WT
+ GNAQ Q209L
+ GNAQ Q209P
GNAQ
FASN
pERK1/2
ERK1/2
HSP90

Figure S2. Major metabolic enzyme expression in NCMs and UM cells. (A) Expression of several key enzymes in glucose uptake, glycolysis, and fatty acid oxidation in NCMs and UM cells were probed by western blot. **(B)** Quantitation of expression of lipogenic enzymes in NCMs and UM cells shown in Figure 1C by densitometry. **(C)** Quantitation of expression of lipogenic enzymes in NCMs and PDXs shown in Figure 1D. **(D)** MP46 cells were treated with siRNA against *GNAQ* for 72 hours. GNAQ and FASN expression were probed by western blot and quantitated by densitometry. β-actin serves as a loading control. Data are shown as mean ± SEM (n=3). *$p<0.05$, **$p<0.01$ **(E)** The protein expression of major lipogenic enzymes in BAP1 wild-type and BAP1-deficient UM cell lines were evaluated by western blot. **(F)** Western blot of FASN in HEK293 *GNAQ*/11 knockout cells transduced with WT or mutant *GNAQ*. β-actin or HSP90 serve as loading controls. NCMs, normal choroidal melanocytes; GLUT1 and 3, glucose transporter; HK1 and 2, hexokinase; PKM2, pyruvate kinase-2; ACSL1, acyl CoA synthetase-1; and CPT1A and 1C, carnitine palmitoyltransferase1.

A

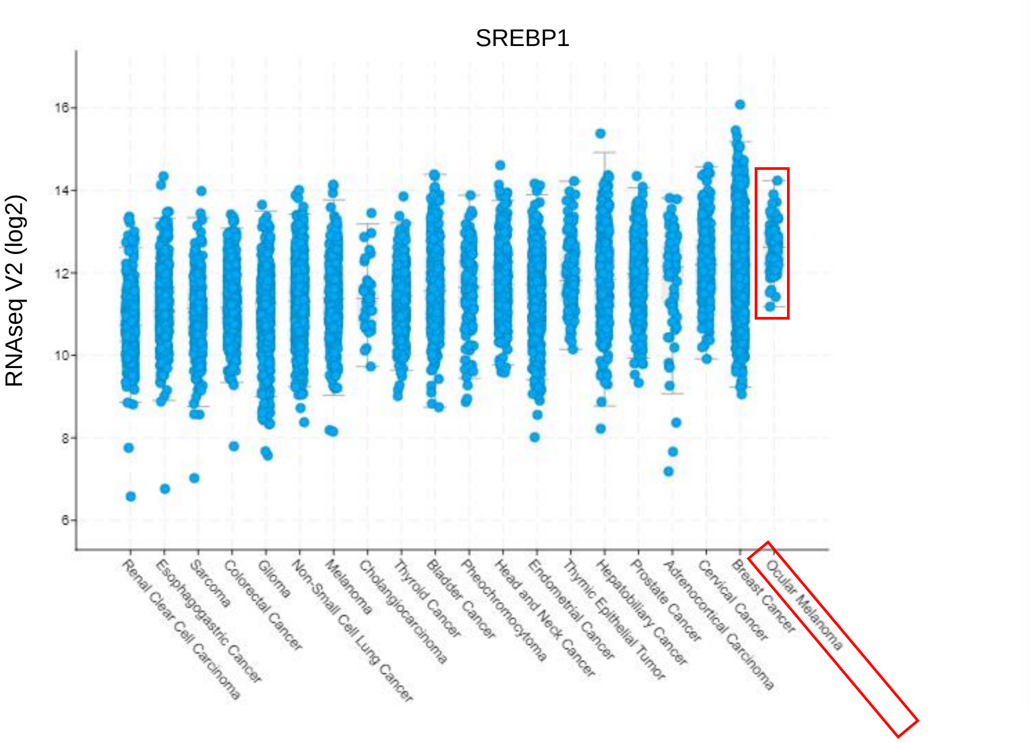
SREBP1
RNAseq V2 (log2)
16
14
12
10
8
6
Renal Clear Cell Carcinoma
Esophagogastric Cancer
Sarcoma
Colorectal Cancer
Glioma
Non-Small Cell Lung Cancer
Melanoma
Cholangiocarcinoma
Thyroid Cancer
Bladder Cancer
Pheochromocytoma
Head and Neck Cancer
Endometrial Cancer
Thymic Epithelial Tumor
Hepatobiliary Cancer
Prostate Cancer
Adrenocortical Carcinoma
Cervical Cancer
Breast Cancer
Ocular Melanoma

B

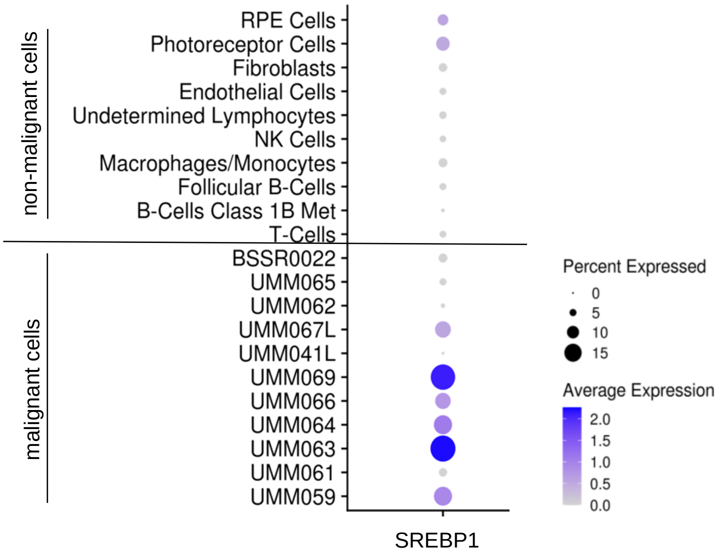
non-malignant cells
RPE Cells
Photoreceptor Cells
Fibroblasts
Endothelial Cells
Undetermined Lymphocytes
NK Cells
Macrophages/Monocytes
Follicular B-Cells
B-Cells Class 1B Met
T-Cells
malignant cells
BSSR0022
UMM065
UMM062
UMM067L
UMM041L
UMM069
UMM066
UMM064
UMM063
UMM061
UMM059
SREBP1
Percent Expressed
0
5
10
15
Average Expression
2.0
1.5
1.0
0.5
0.0

Figure S3. Expression of SREBP1 in UM patient samples. (A) Gene expression of SREBP1 in various human cancer types. Data were derived from a TCGA dataset and analyzed through the Firebrowse web resource (http://firebrowse.org/, accessed on 24 February 2021). Box plots display FASN and SREBP1 levels measured as RSEM log2 across various cancers. Red box indicates uveal melanoma samples (UVM). **(B)** The average expression of SREBP1 in non-malignant and malignant cells from UM tumors**.**

A

Cell Line	Sources	Mutation Status (GNAQ)
92.1	Primary	Q209L
OMM1.3	Liver met	Q209L
UM001	Liver met	Q209L
UM004	Orbital met	Q200P

B

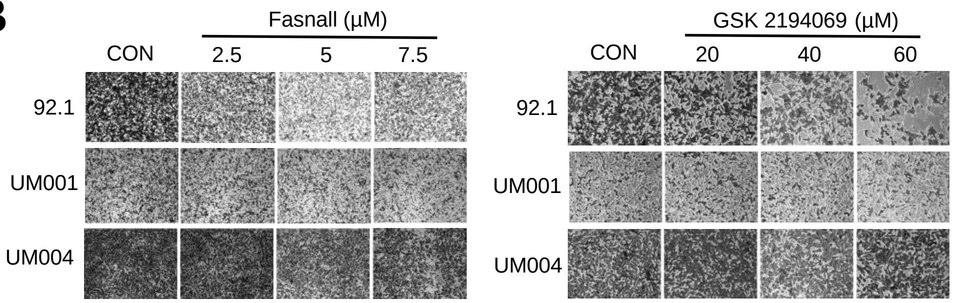

C

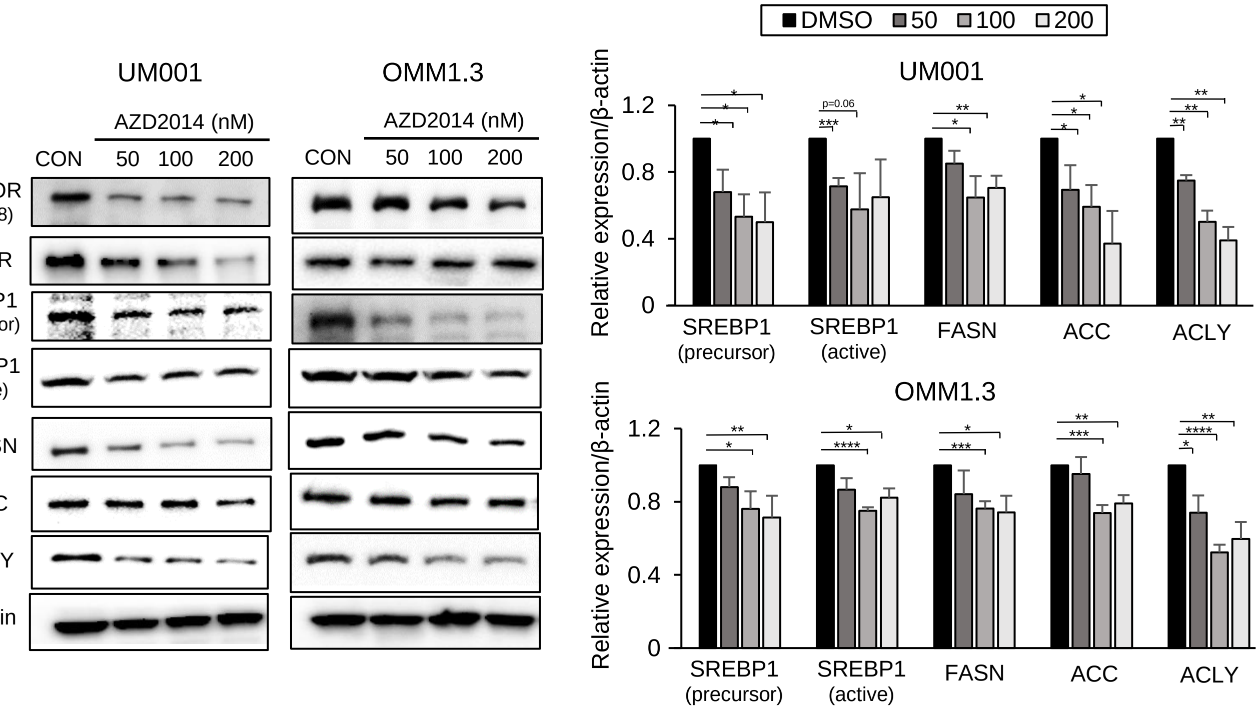

D

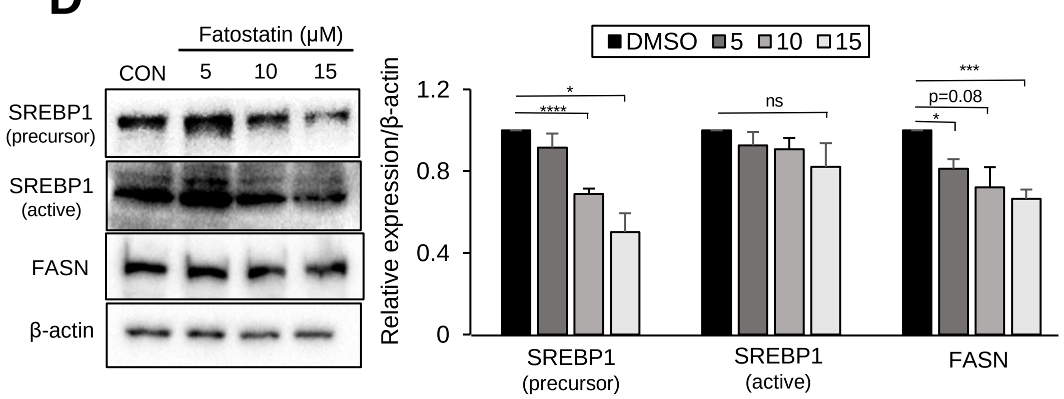

Figure S4. mTOR-SREBP1 axis regulates FASN expression in UM cells.

(A) GNAQ mutational status of UM cells. **(B)** 92.1, UM001, and UM004 cells were treated with Fasnall (0, 2.5, 5 and 7.5 μM) for 4 days or GSK 2194069 (0, 20, 40 and 60 μM) for 3 days. Cell viability was measured by crystal violet staining. Representative images from four biological replicates are shown. **(C)** UM001 and OMM1.3 cells were treated with AZD2014 (0, 50, 100 and 200 nM) for 48 hours. Activation of mTOR and expression of SREBP1, FASN, ACC and ACLY were detected by western blot. **(D)** UM001 cells were treated with Fatostatin (0, 5, 10 and 15 μM) for 24 hours. Reduction of SREBP1 and FASN expression were probed by western blot. β-actin serves as a loading control. Data are shown as mean ± SEM (n=3). ns, not significant,

*$p<0.05$, **$p<0.01$, ***$p<0.001$ and ****$p<0.0001$ unpaired t-test.

A

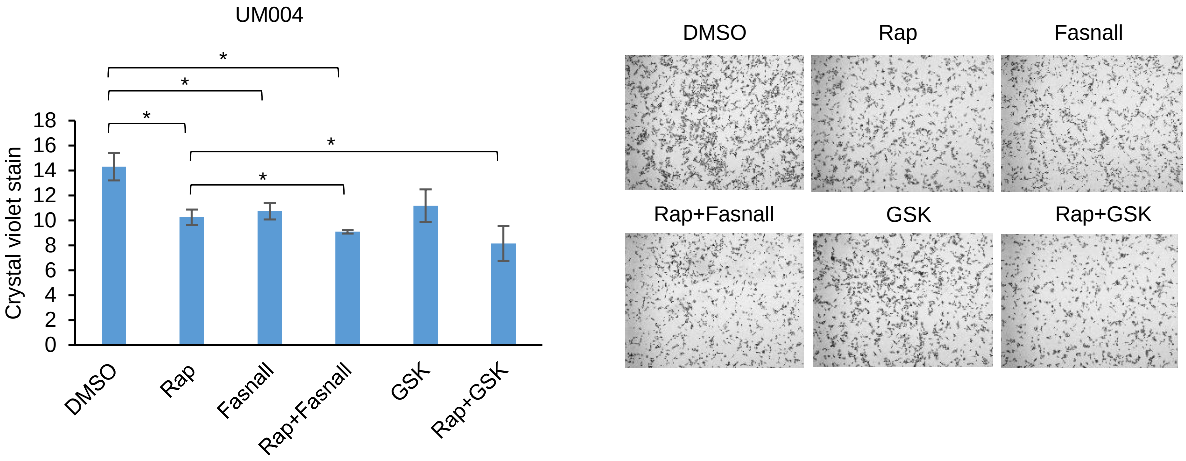

B

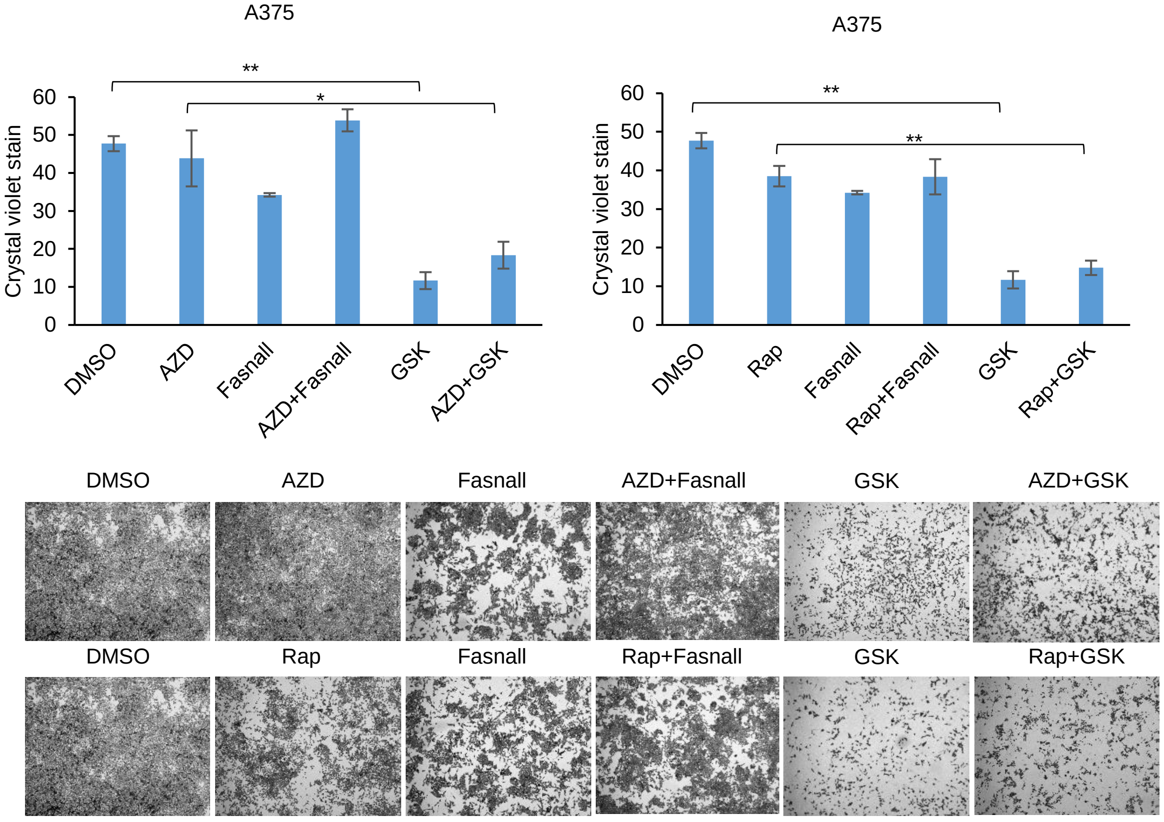

Figure S5. FASN and mTOR inhibitor effects in non-GNAQ mutant and UM cell lines. (A) UM004 UM cells and **(B) t**he A375 (non-GNAQ mutant) cutaneous melanoma cell line were treated with FASN inhibitors (5 µM Fasnall or 40 µM GSK2194069) and/or mTOR inhibitors (200 nM AZD2014 or 100 nM Rapamycin) for 72 hours. Cell viability was determined by crystal violet staining. Data are shown as mean ± SEM (n=3). *p<0.05, **p<0.01 unpaired t-test. Representative crystal violet images are also shown.

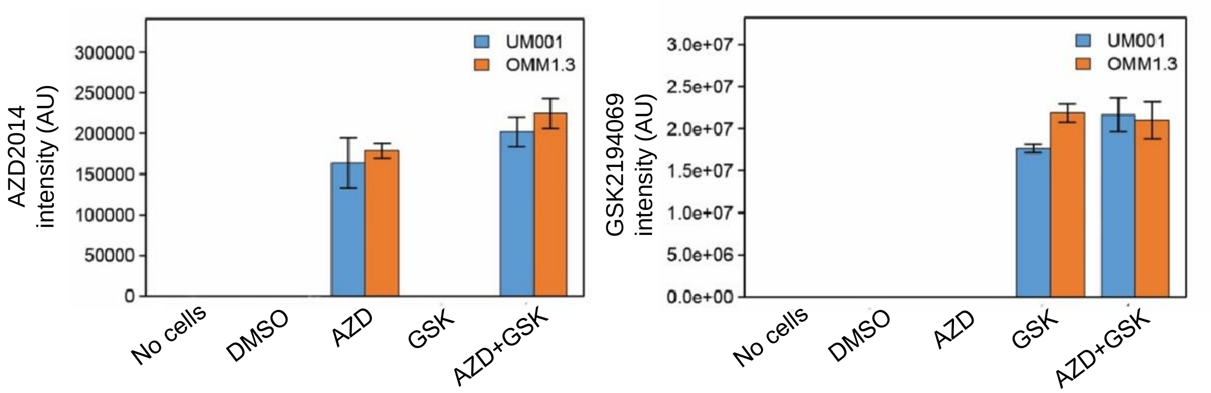

Figure S6. Uptake of FASN and mTOR inhibitors by UM cells. UM001 and OMM1.3 cells were incubated in the presence of ^{13}C-glucose for 4 and 24 hours along with GSK2194069 (40 μM) with or without AZD2014 (200 nM). Peak area intensity for AZD2014 and GSK2194069 in cells as determined by LC-MS/MS. Data are shown as mean ± SEM (n=6). Student t-test. Uptak.

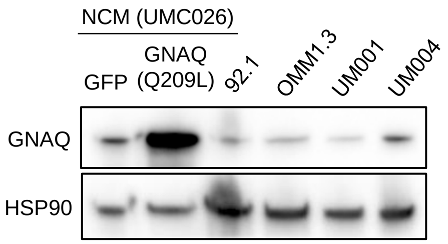

Figure S7. GNAQ levels in melanocyte model and UM cell lines. Western blot was performed to identify GNAQ levels between UMC026 cells transduced with *GNAQ* Q209L with UM cell lines, 92.1, OMM1.3, UM001, and UM004.

Figure 1A

GNAQ (box)

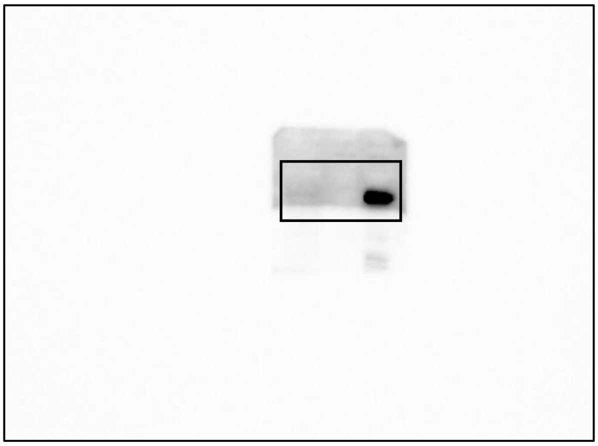

β-actin (box)

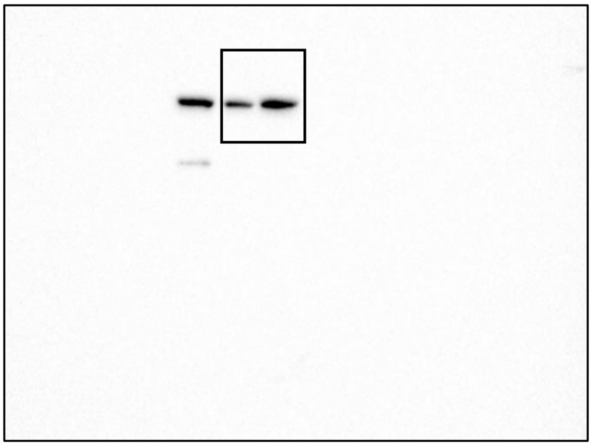

pERK1/2 (box)

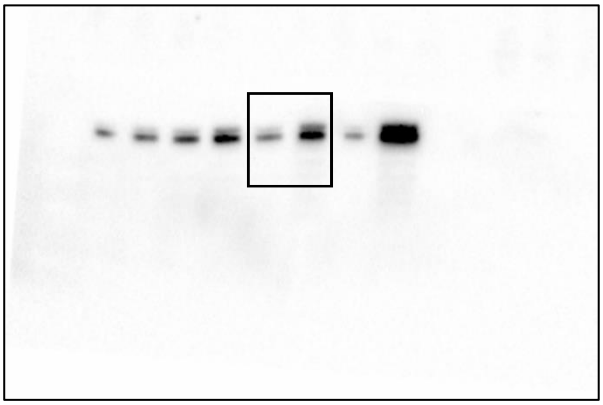

ERK1/2 (box)

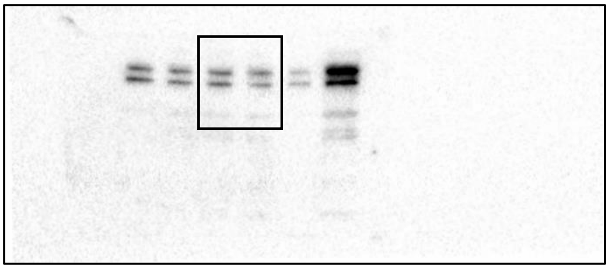

HSP90 (box)

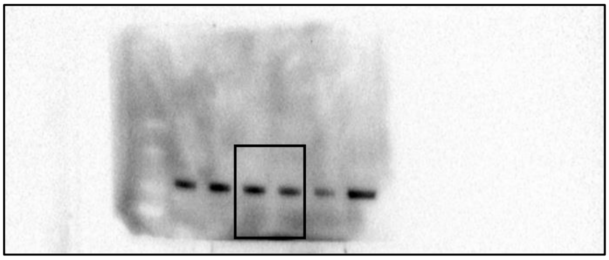

Figure 1C

ACLY (box)

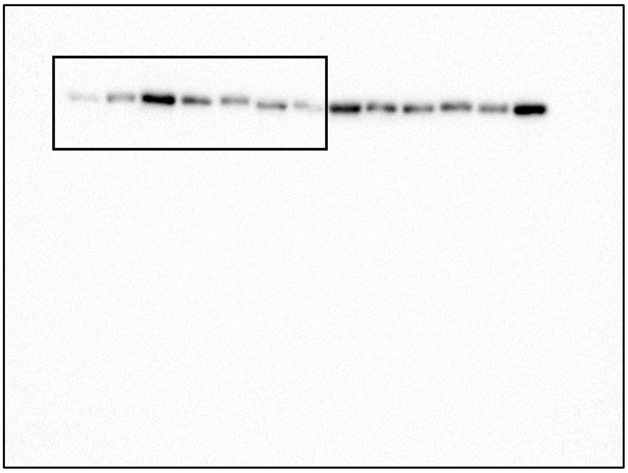

ACC (box)

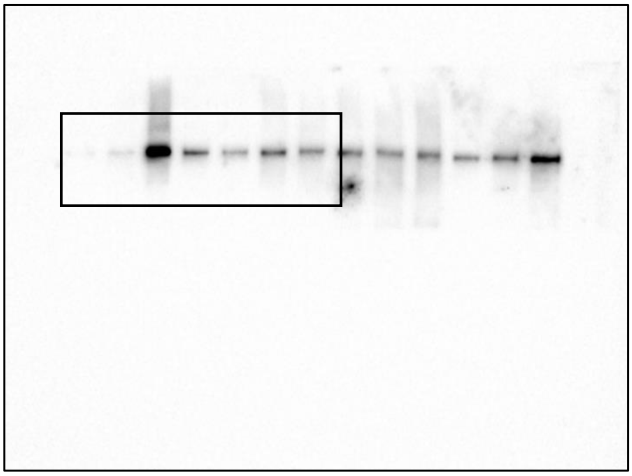

FASN (box)

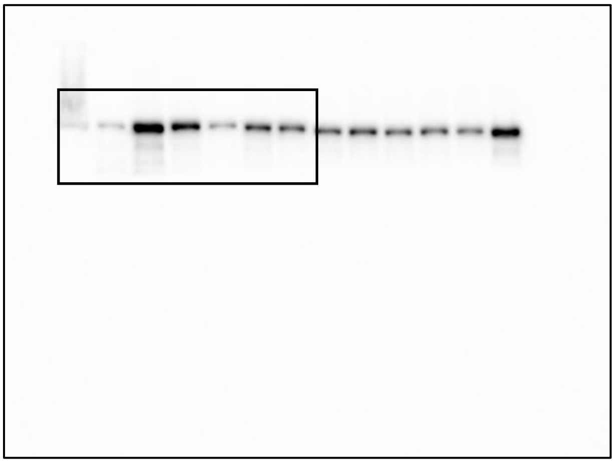

β-actin (box)

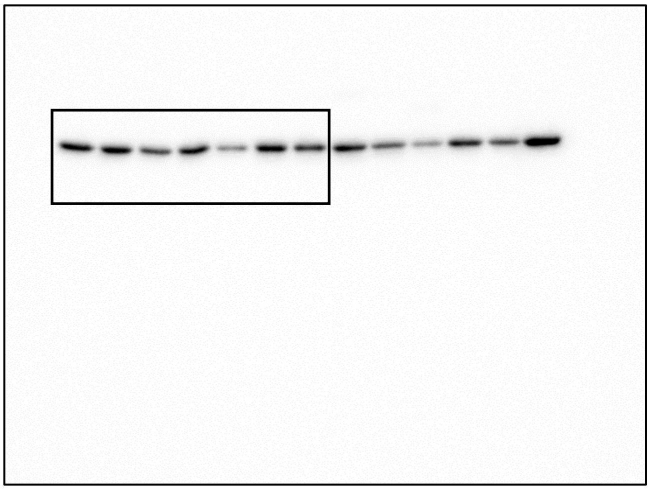

Figure 1C

SREBP1 _precursor (box)

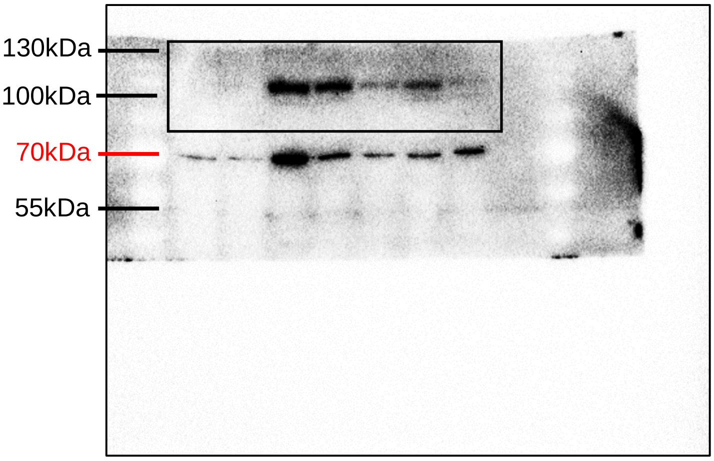

SREBP1 _active (box)

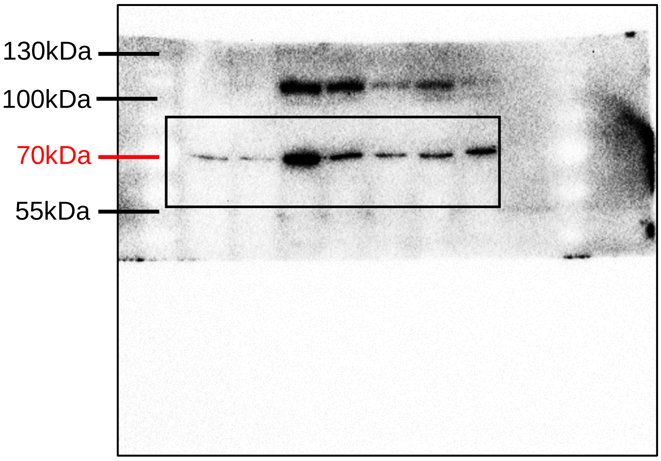

Figure 1D

ACLY (box)

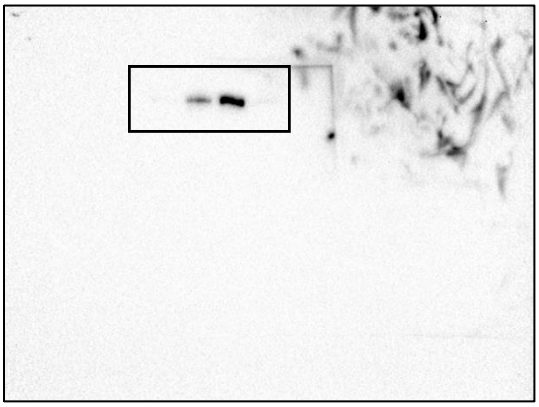

ACC (box)

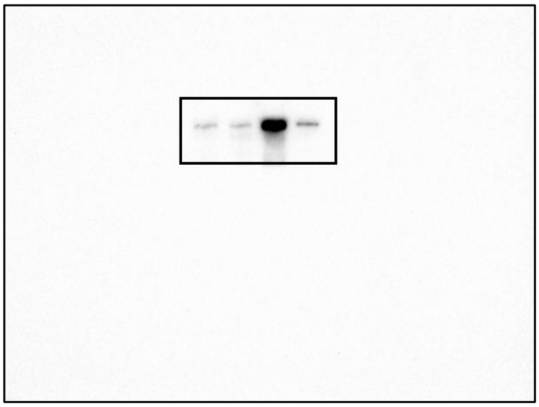

SREBP1 _precursor (box)

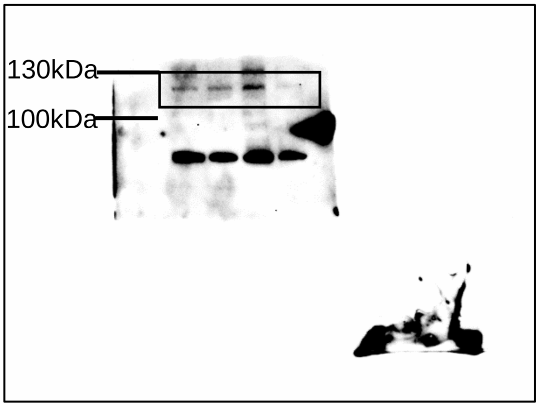

SREBP1 _active (box)

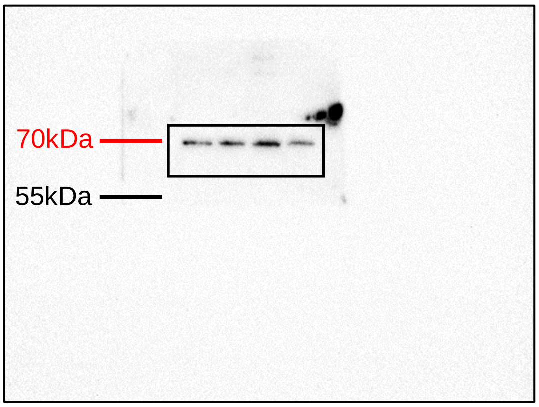

Figure 1D

FASN (box)

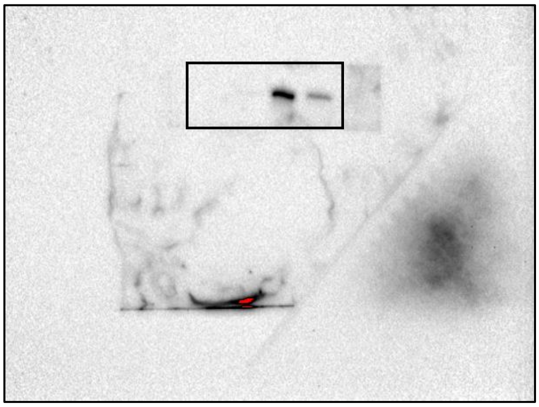

β-actin (box)

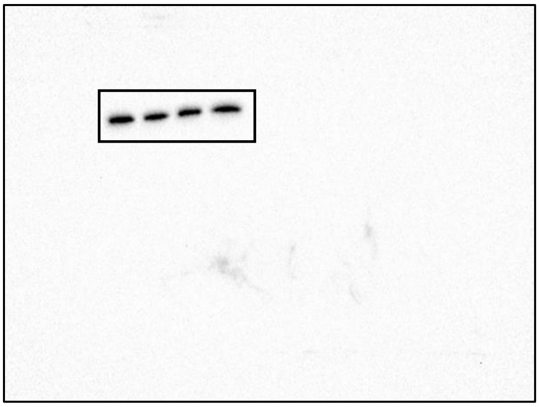

Figure 1E

GNAQ (box)

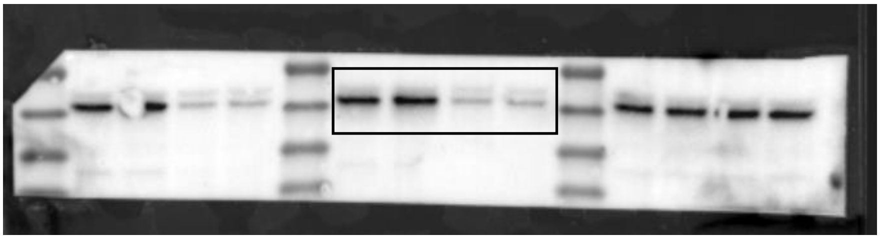

FASN (box)

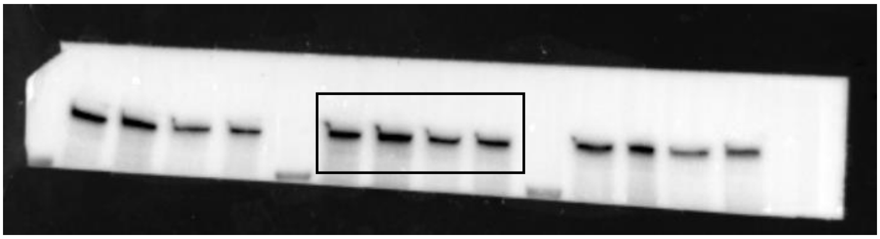

β-actin (box)

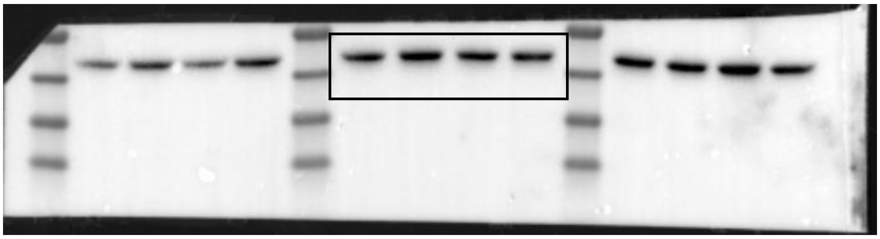

Figure 3B

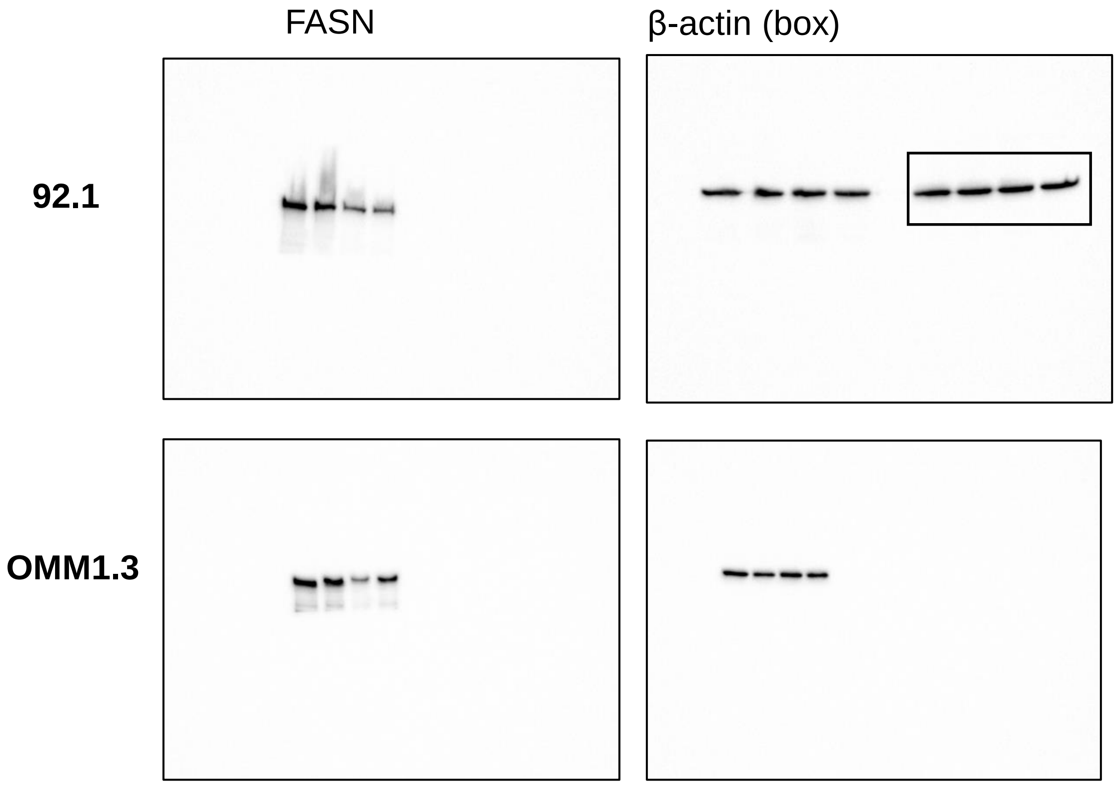

Figure 3D

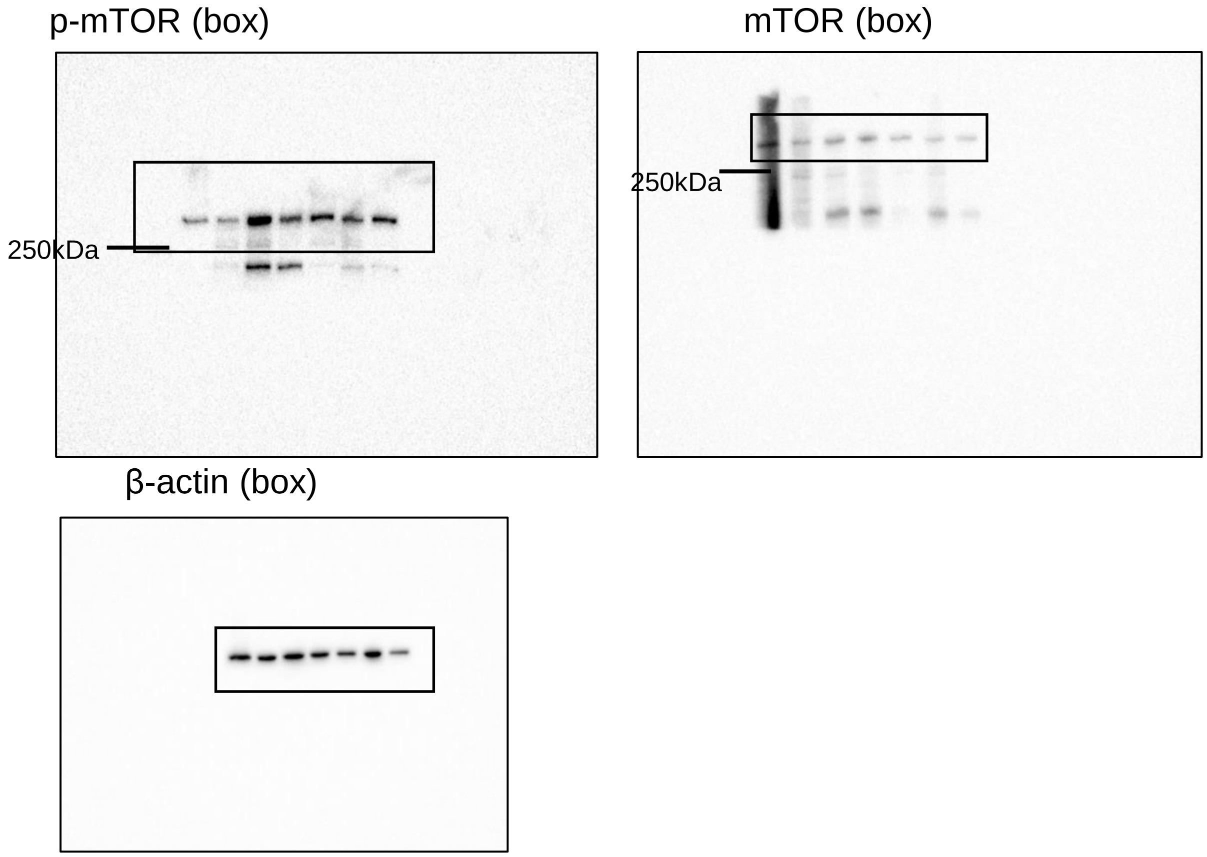

Figure 4B (UM001, left panel) : Fas

p-ACC (box)

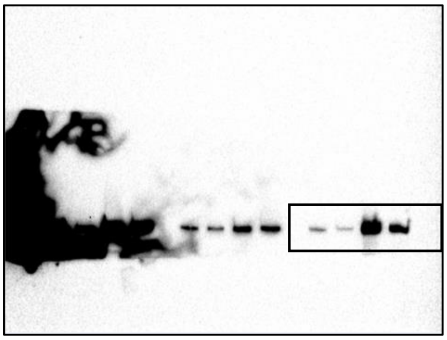

Aurora-A (box)

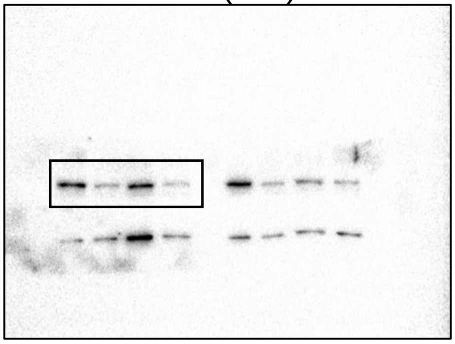

Cyclin B (box)

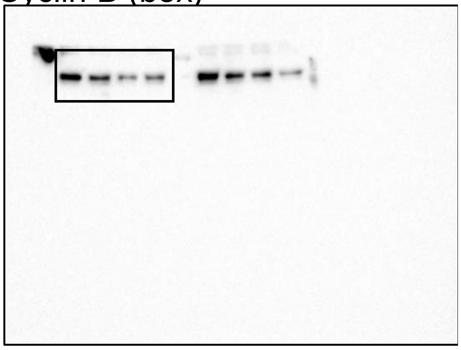

PLK1 (box)

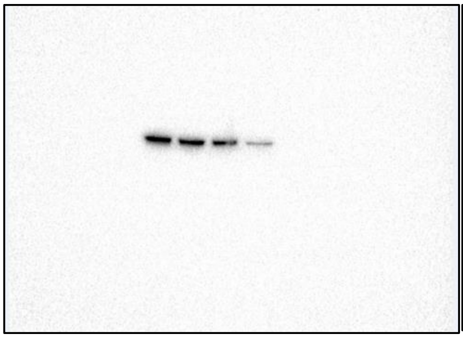

p-RB (box)

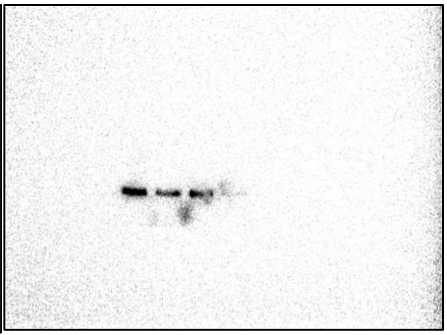

β-actin (box)

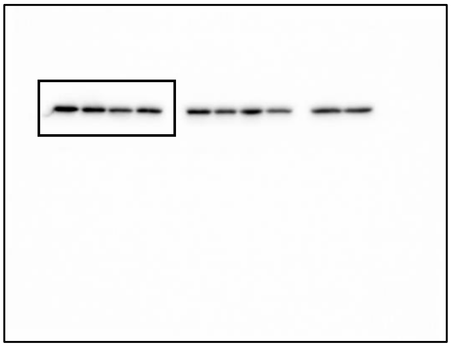

HSP90 (box)

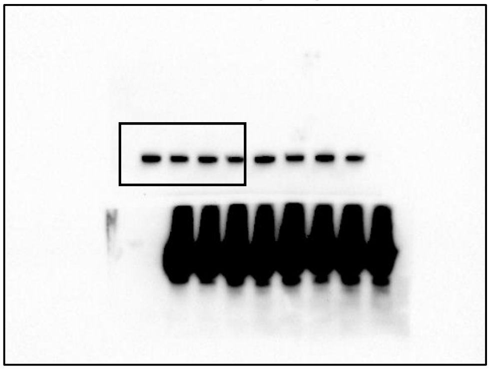

S6 (box)

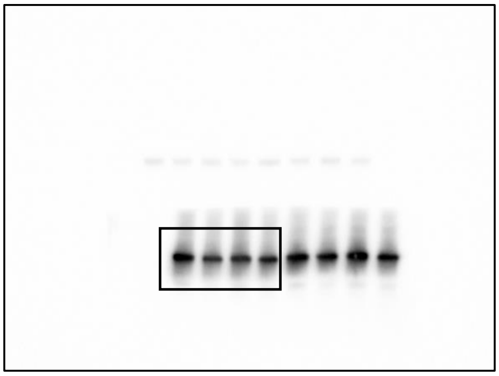

S6 (p235/236) (box)

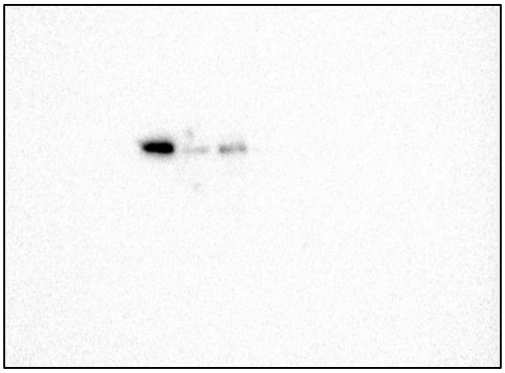

S6 (p240/244) (box)

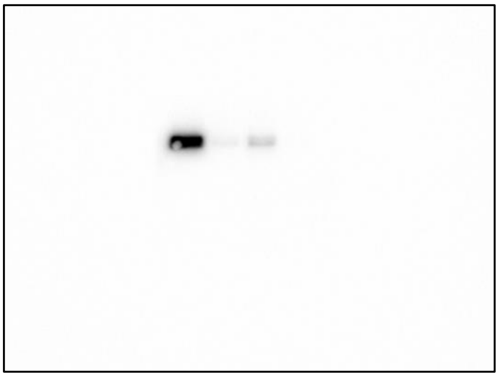

Figure 4B (UM001, right panel) : GSK

p-ACC (box)

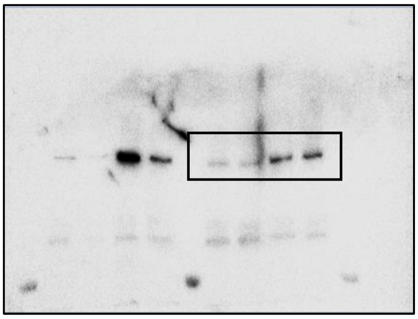

Aurora-A (box)

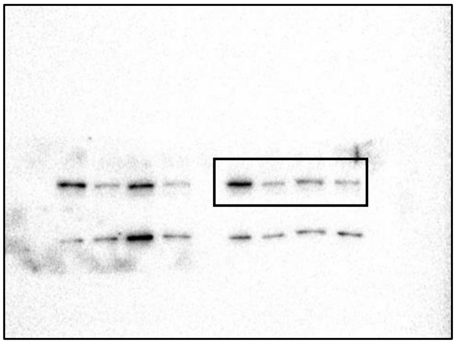

Cyclin B (box)

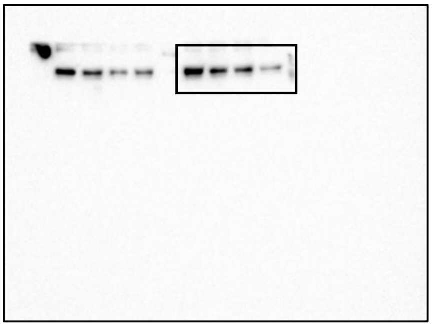

PLK1 (box)

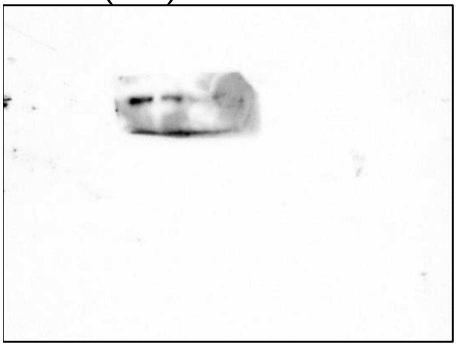

p-RB (box)

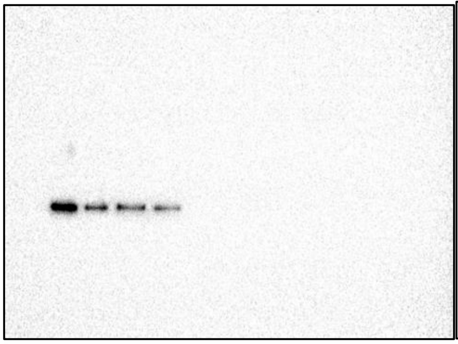

β-actin (box)

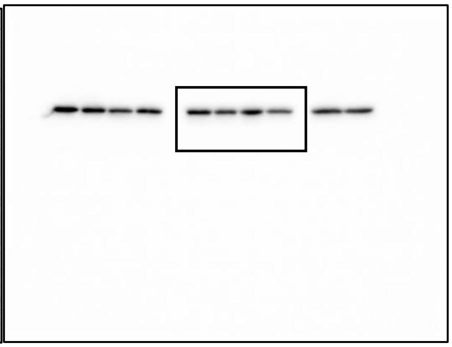

S6 (p235/236) (box)

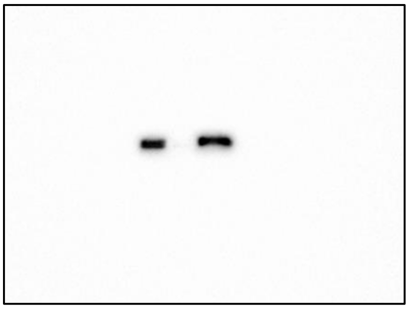

S6 (p240/244) (box)

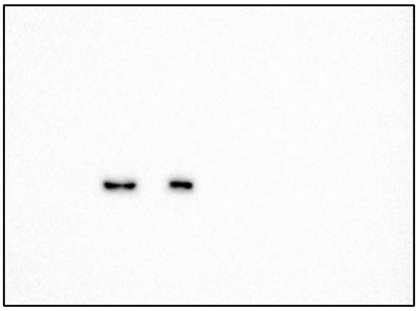

HSP90 (box)

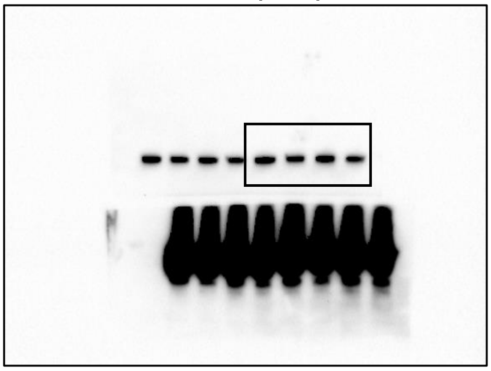

S6 (box)

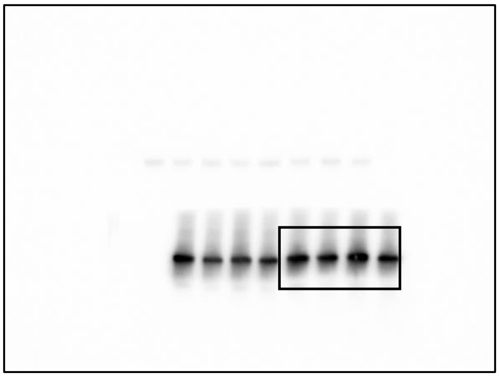

Figure 4B (UM004)

p-acc

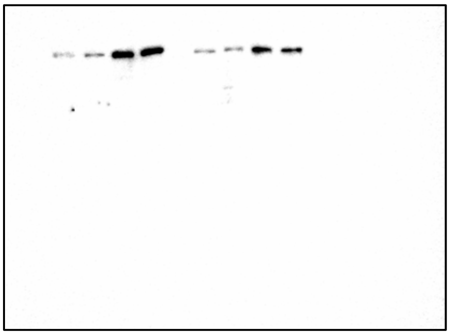

Aurora A

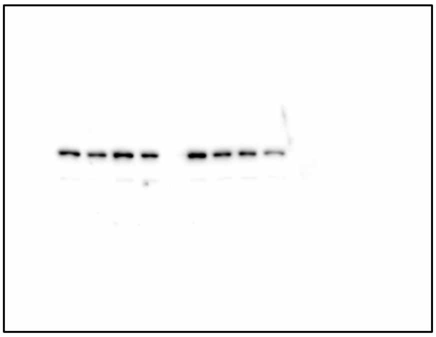

Cyclin B1 (Box)

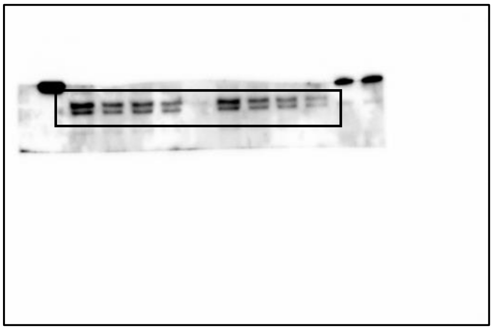

PLK1 (Box)

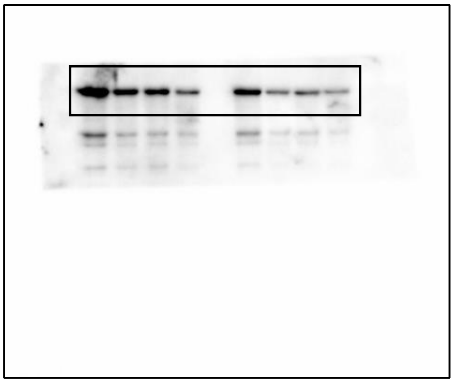

p-RB

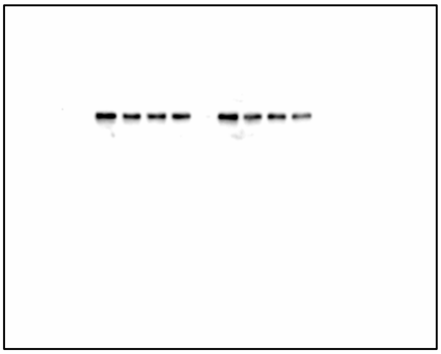

p-S6 235/236

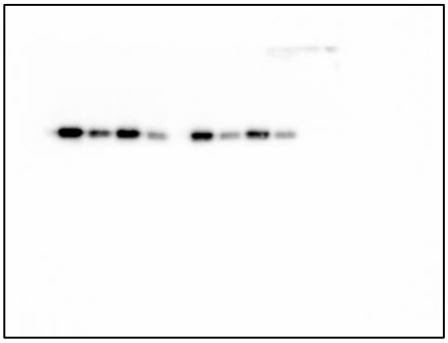

p-S6 240/244

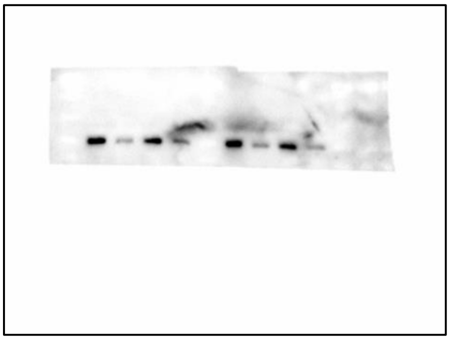

Actin

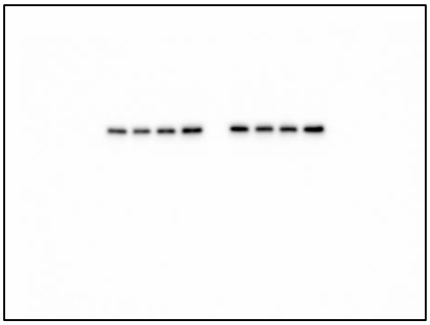

HSP90 (Box)

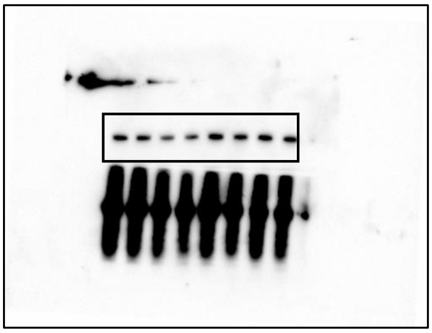

S6 (Box)

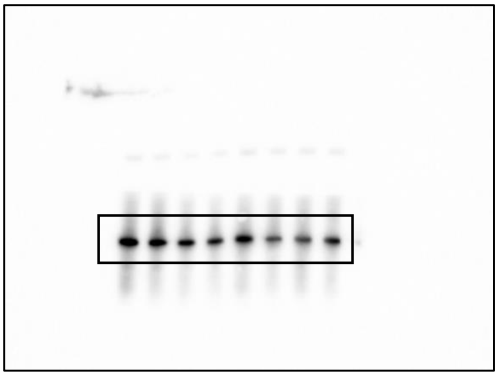

Supplementary Figure S2A

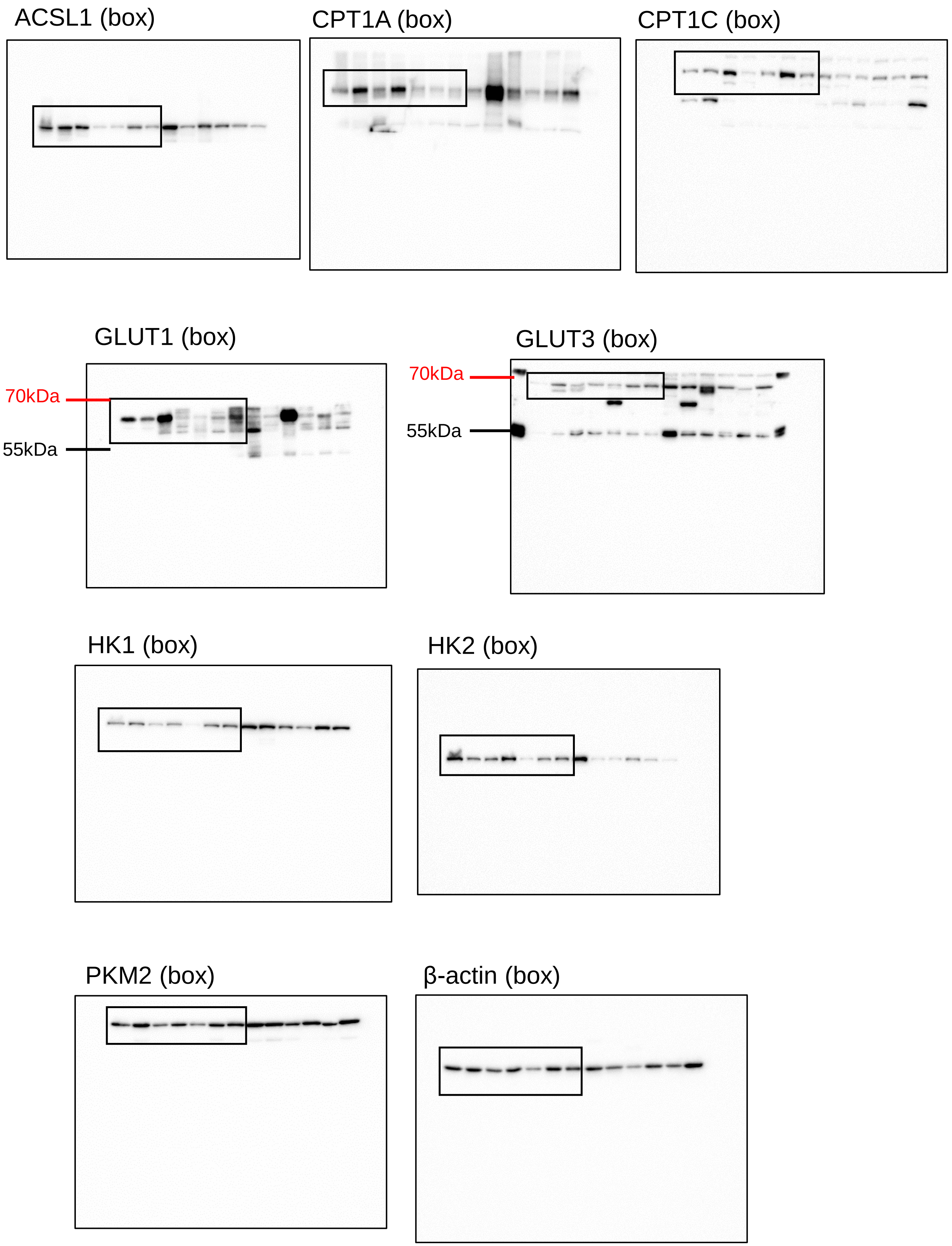

Supplementary Figure S2E

BAP1 (box)

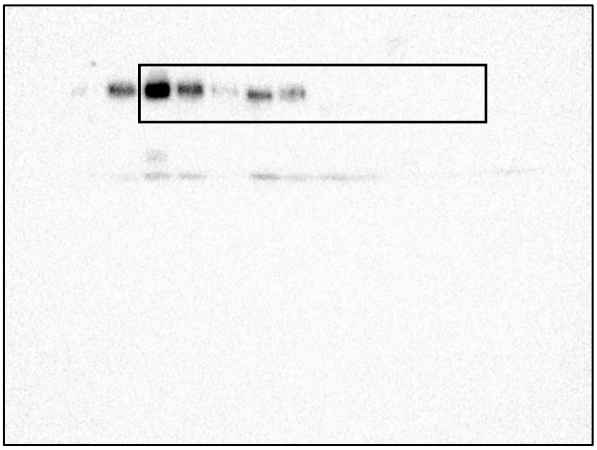

ACLY (box)

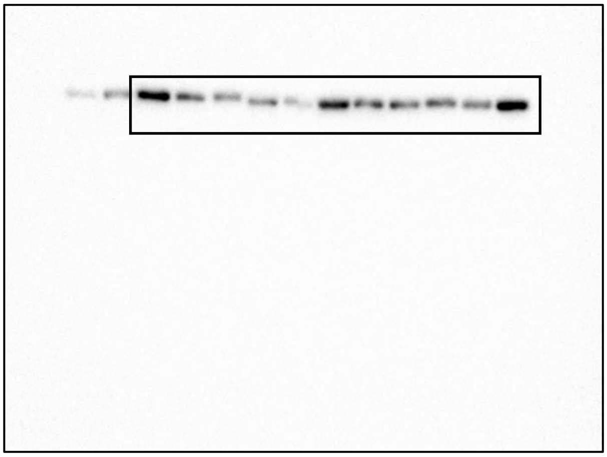

ACC (box)

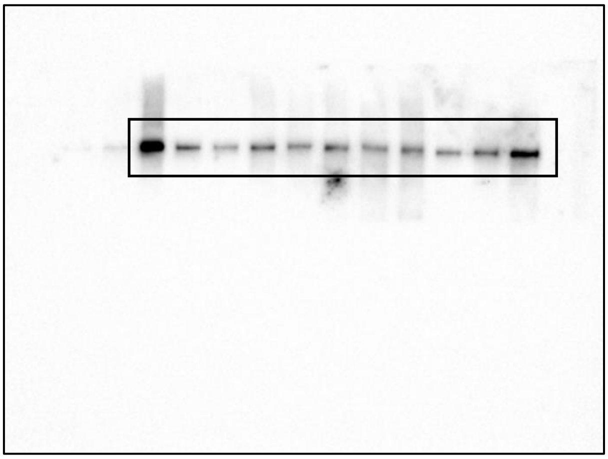

FASN (box)

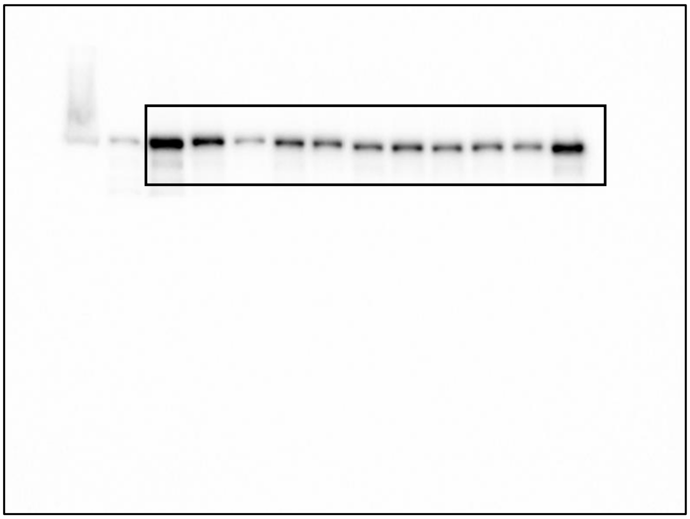

β-actin (box)

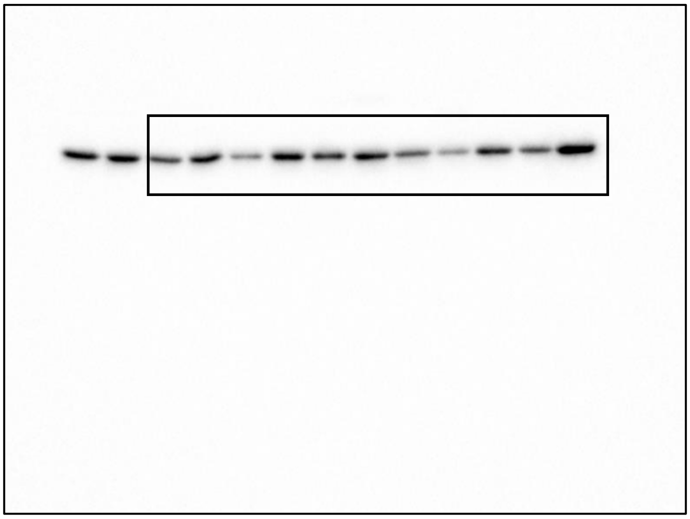

Supplementary Figure S2F

GNAQ (box)

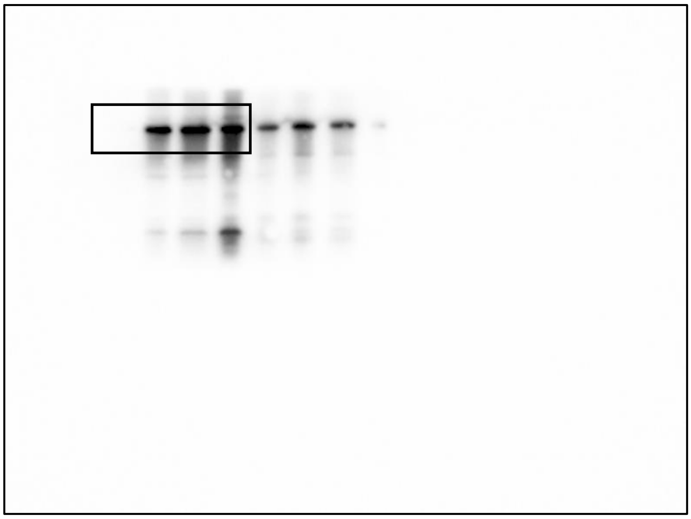

FASN (box)

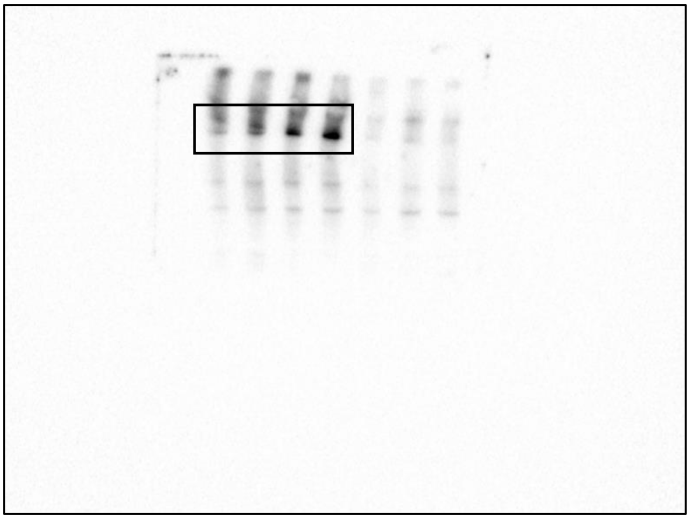

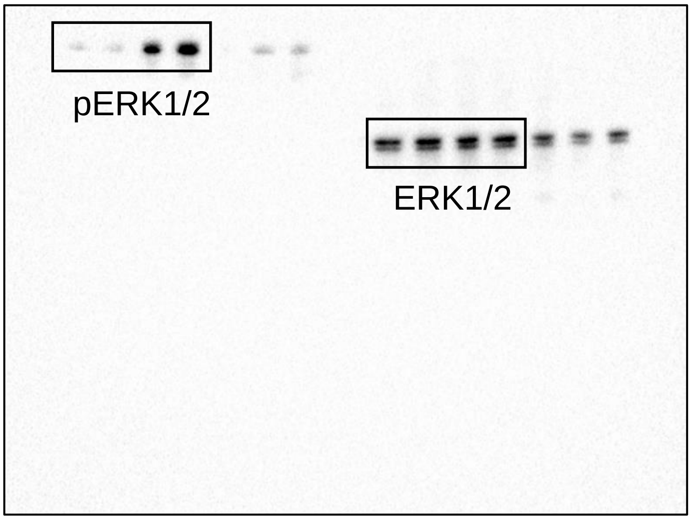

HSP90 (box)

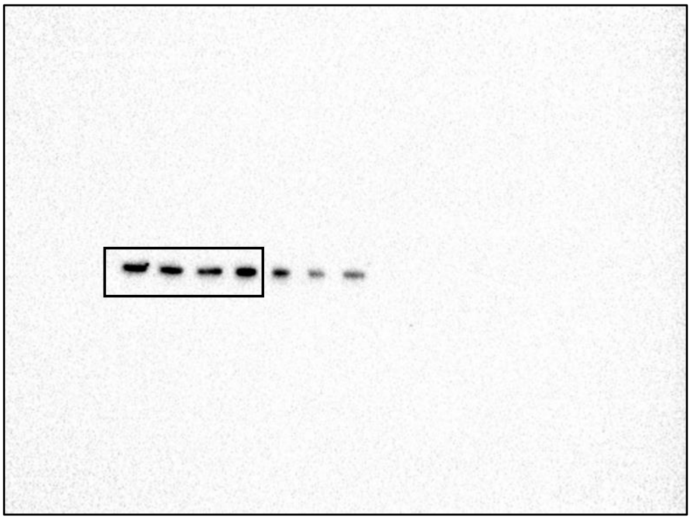

Supplementary Figure S4C (UM001)

p-mTOR(box)

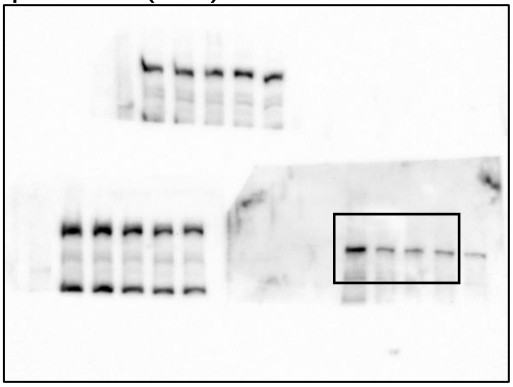

mTOR(box)

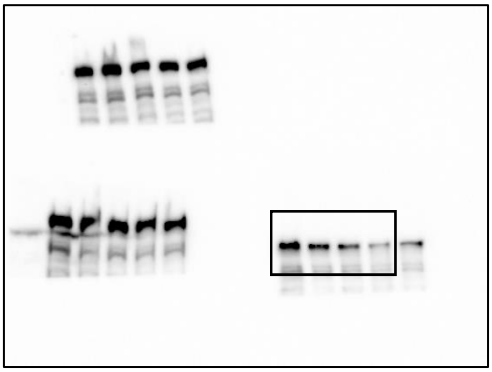

SREBP1 _precursor (box)

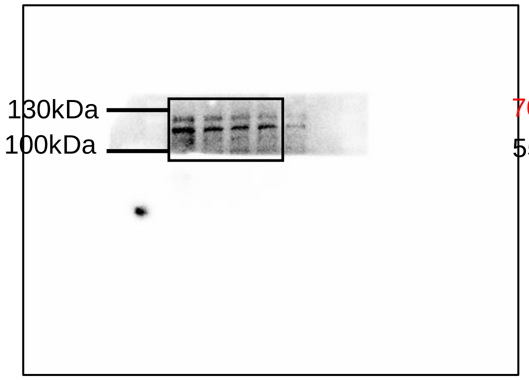

SREBP1 _active (box)

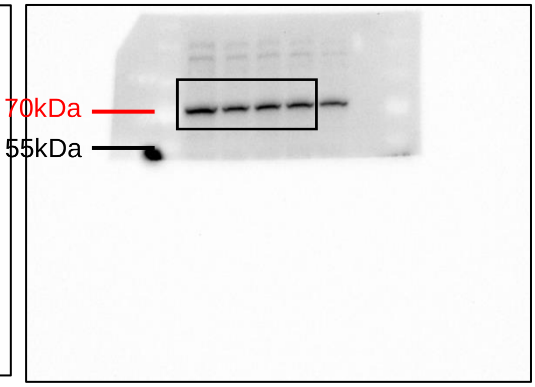

ACC (box)

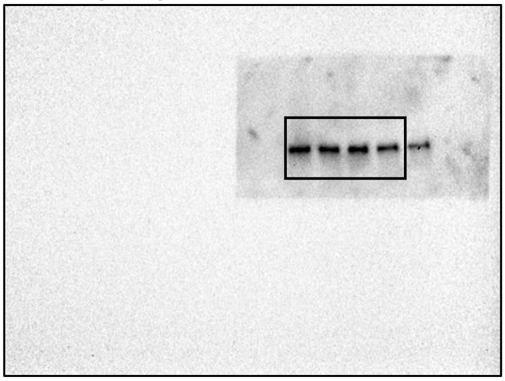

ACLY (box)

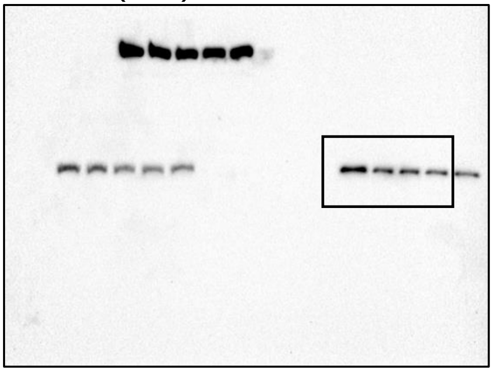

FASN (box)

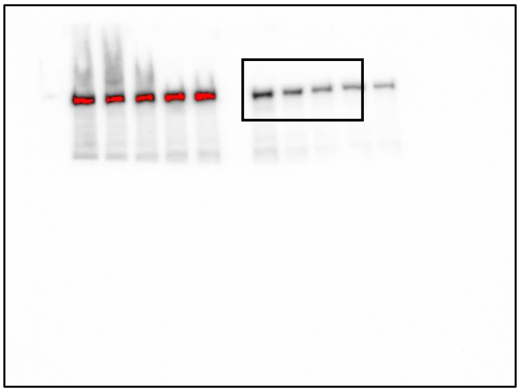

β-actin (box)

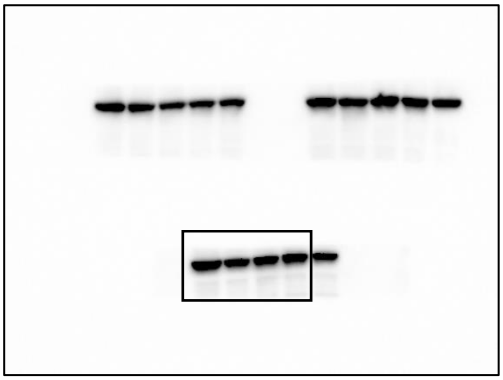

Supplementary Figure S4C (OMM1.3)

p-mTOR(box)

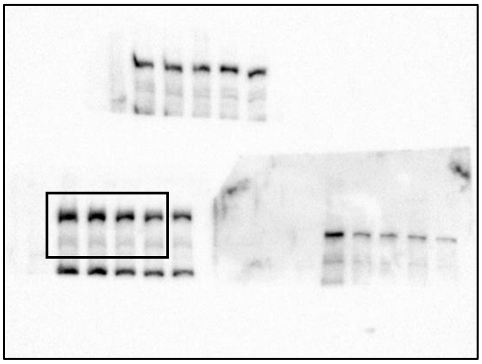

mTOR(box)

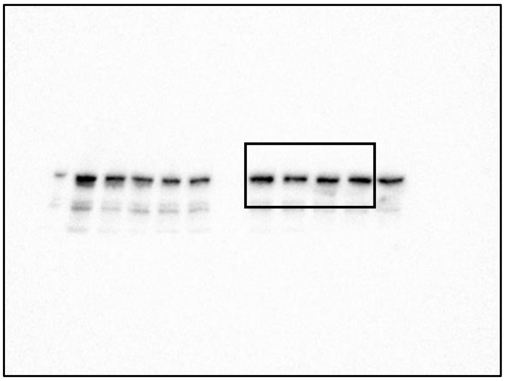

SREBP1 _active (box)

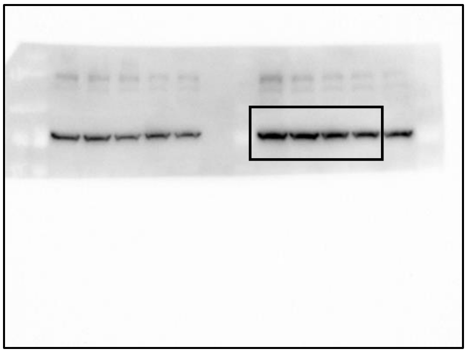

FASN (box)

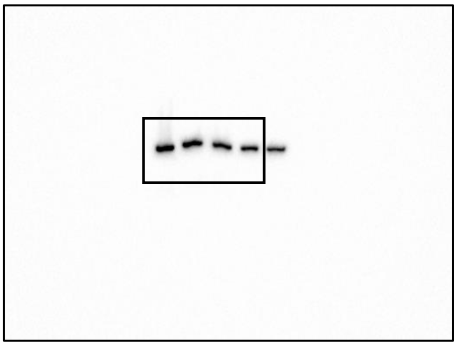

ACLY (box)

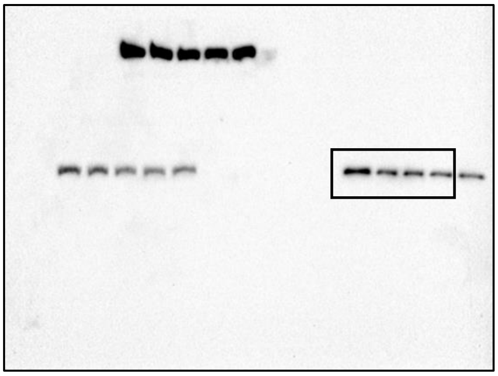

β-actin (box)

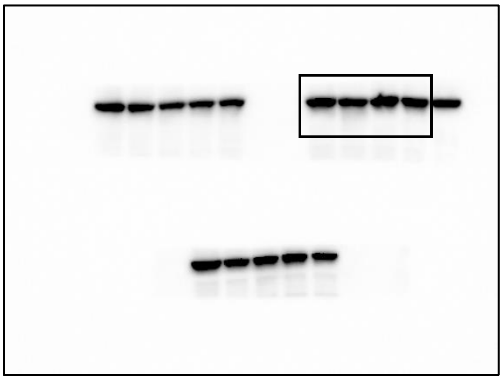

SREBP1 _precursor (box)

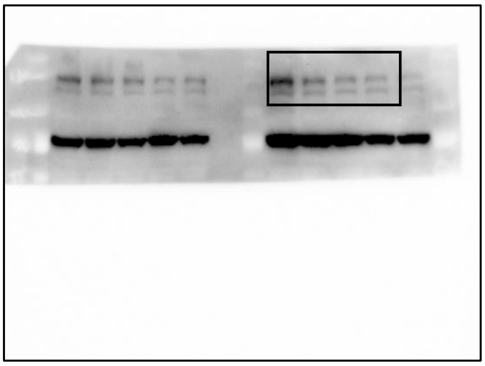

ACC (box)

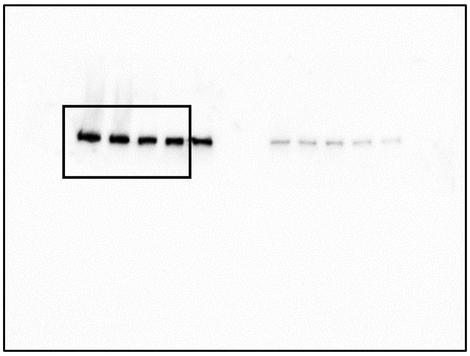

Supplementary Figure S4D (UM001)

SREBP1 _precursor (box)

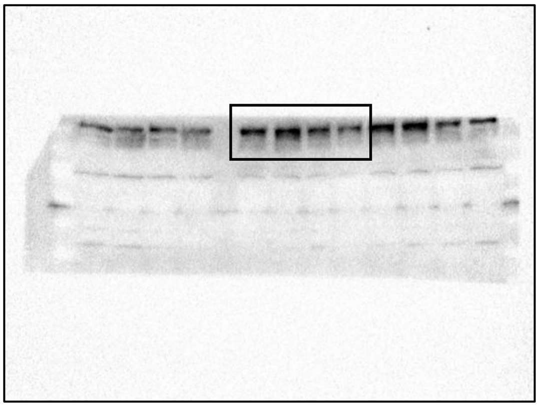

SREBP1 _active (box)

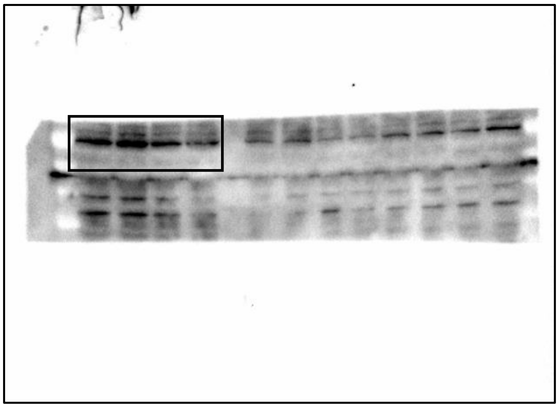

FASN (box)

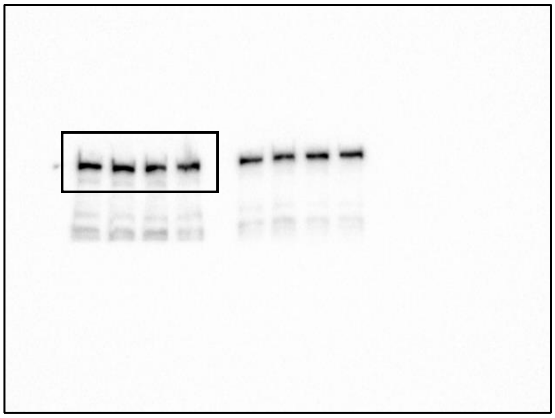

β-actin (box)

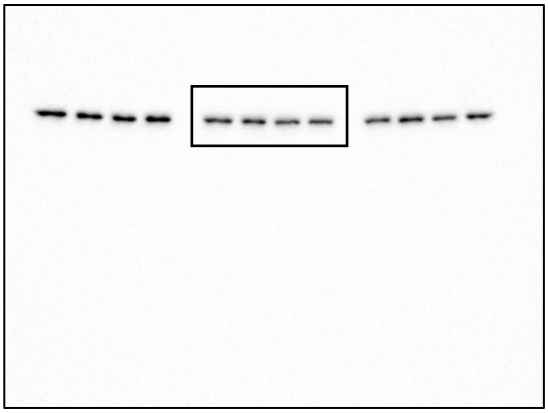

Supplementary Figure S7

GNAQ (box)

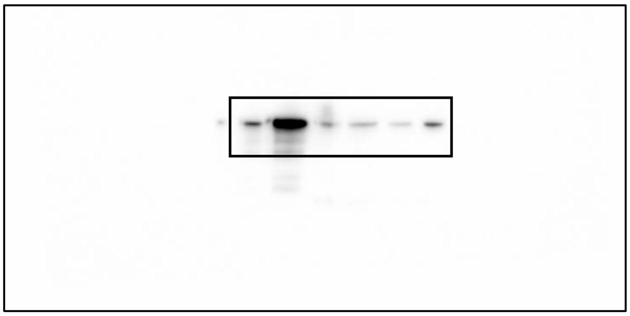

HSP90 (box)

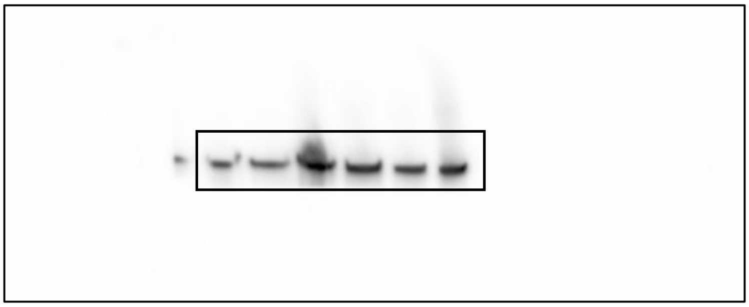

Figure S8. Original western blots.
